# Advancing Soil Organic Carbon Prediction: A Comprehensive Review of Technologies, AI, Process‐Based and Hybrid Modelling Approaches

**DOI:** 10.1002/advs.202504152

**Published:** 2025-06-25

**Authors:** Zijuan Ding, Ke Liu, Sabine Grunwald, Pete Smith, Philippe Ciais, Bin Wang, Alexandre M.J.‐C. Wadoux, Carla Ferreira, Senani Karunaratne, Narasinha Shurpali, Xiaogang Yin, Dale Roberts, Oli Madgett, Sam Duncan, Meixue Zhou, Zhangyong Liu, Matthew Tom Harrison

**Affiliations:** ^1^ Tasmanian Institute of Agriculture University of Tasmania Newnham Drive Launceston TAS 7249 Australia; ^2^ Pedometrics Landscape Analysis & GIS Laboratory Soil Water and Ecosystem Sciences Department University of Florida Gainesville FL 32611‐0290 USA; ^3^ Institute of Biological and Environmental Sciences University of Aberdeen Cruickshank Building, St Machar Drive Aberdeen AB24 3UU UK; ^4^ Laboratoire des Sciences du Climat et de l'Environnement CEA‐CNRS‐UVSQ Gif‐sur‐Yvette 91190 France; ^5^ NSW Department of Primary Industries Wagga Wagga Agricultural Institute Wagga Wagga NSW 2650 Australia; ^6^ Hawkesbury Institute for the Environment Western Sydney University Richmond NSW 2753 Australia; ^7^ Gulbali Institute for Agriculture Water and Environment Charles Sturt University Wagga Wagga NSW 2650 Australia; ^8^ LISAH Univ Montpellier AgroParisTech INRAE IRD L'Institut Agro Montpellier 34060 CEDEX 2 France; ^9^ Polytechnic Institute of Coimbra Applied Research Institute Rua da Misericórdia Lagar dos Cortiços—S. Martinho do Bispo Coimbra 3045–093 Portugal; ^10^ CSIRO Agriculture and Food Ngunnawal Country Clunies Ross Street Black Mountain ACT 2601 Australia; ^11^ Natural Resources Institute Finland (LUKE) Halolantie 31 A Maaninka 71750 Finland; ^12^ College of Agronomy and Biotechnology China Agricultural University and Key Laboratory of Farming System Ministry of Agriculture and Rural Affairs of China Beijing 100193 China; ^13^ Farmlab Pty. Ltd. 122 Faulkner St Armidale NSW 2350 Australia; ^14^ College of Agriculture Yangtze University Hubei Province 434023 China

**Keywords:** biogeochemical model, data‐fusion, deep learning, hybrid approaches, machine learning, remote sensing, soil carbon prediction

## Abstract

Measurement, monitoring, and prediction of soil organic carbon (SOC) are fundamental to supporting climate change mitigation efforts and promoting sustainable agricultural management practices. This review discusses recent advances in methodologies and technologies for SOC quantification, including remote sensing (RS), proximal soil sensing (PSS), artificial intelligence (AI) for SOC modelling (in particular, machine learning (ML) and deep learning (DL)), biogeochemical modelling, and data fusion. Integrating data from RS, PSS, and other sensors usually leads to good SOC predictions, provided it is supported by careful calibration, validation across diverse pedo‐climatic and land management, and the use of data processing and modelling frameworks. We also found that the accuracy of AI‐driven SOC prediction improves when RS covariates are included. Although DL often outperforms classical ML, there is no single best AI algorithm. By incorporating simulated outputs from biogeochemical model as additional training data for AI, causal relationships in SOC turnover can be incorporated into empirical modelling, while maintaining predictive accuracy. In conclusion, SOC prediction can be enhanced through 1) integrating sensing technologies, 2) applying AI, notably DL, 3) addressing biogeochemical model limitations (assumptions, parameterization, structure), 4) expanding SOC data availability, 5) improving mathematical representation of microbial influences on SOC, and 6) strengthening interdisciplinary cooperation between soil scientists and model developers.

## Graphical abstract

This review highlights advances in soil organic carbon (SOC) quantification using remote sensing, proximal soil sensing, AI (ML and DL), and biogeochemical modelling. Integrating diverse data sources and models improves SOC prediction accuracy. Key priorities include enhancing data availability, refining models, incorporating microbial processes, and fostering interdisciplinary collaboration to support sustainable agriculture and climate mitigation.

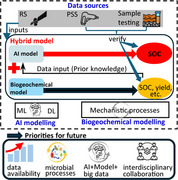

## 1 Introduction

The terrestrial biosphere comprised the world's second largest carbon pool, with organic carbon stored in vegetation and soils thought to be around double the amount of carbon in the atmosphere.^[^ ^1^ ^]^ Rates of soil organic carbon (SOC) loss have accelerated significantly over the past two centuries, with some authors suggesting that agriculture has depleted 133 Pg C from the top two meters of soil.^[^ ^2^ ^]^ The scale of the terrestrial biosphere means that even minute perturbations in SOC have disproportionate effects on atmospheric CO_2_ concentrations and thus global warming (or lack thereof).^[^ ^3^ ^]^ Quantification of SOC stocks at large scales is essential not only for assessing global climate change and informing policy decisions, but also for evaluating soil health and guiding sustainable agricultural practices.^[^ ^4^ ^]^ While methods for SOC prediction abound, accuracy of SOC estimates is generally proportional to cost and inversely proportional to scale of assessment. This trade‐off is particularly evident when comparing different methodological approaches. For example, remote sensing (RS) can be used to infer surface SOC over large areas at a relatively low cost, although with lower accuracy compared to physical soil coring methods.^[^ ^5^ ^]^ Despite its limitations in precision, RS offers significant advantages by enabling the extraction of key environmental and spatial variables linked to soil carbon dynamics, such as land cover, vegetation type, soil texture, parent materials, topography, and climate.^[^ ^6^ ^]^ These factors collectively influence the SOC dynamics by regulating carbon inputs, stabilization mechanisms, and microbial activity.

Inclusion of such covariates improves the accuracy of modeled SOC. Advances in RS technology have introduced high‐resolution imagery, expanded spectral bands, and higher satellite revisit frequencies. These advancements provide data sources for SOC prediction based on artificial intelligence (AI) and biogeochemical models, provided proper calibration and workflows are implemented.^[^ ^7^ ^]^ These frameworks can be coupled with data pertaining to climate, land use and soil properties to forecast SOC in response to a change in soil management practices.^[^ ^8^ ^]^ In terms of AI and biogeochemical modelling, SOC accuracy can improve using multi‐model ensembles, with the multi‐model median thought to provide an intuitive measure of overall performance.^[^ ^9^ ^]^ However, such studies require intensive capability compared to running a single model, such that ensemble studies are generally often implemented in collaborative initiatives such as in the Agricultural Modeling Intercomparison Project.^[^ ^10^ ^]^ A gap remains in integrating biogeochemical models (which use process‐based models to predict SOC deterministically) with AI‐based models, which rely on large datasets and predominantly produce statistical results. Bridging this gap could enhance SOC prediction accuracy by leveraging the strengths of both approaches, although training data, calibration, and methods for integrating approaches are an active area of research.

Here we review techniques for SOC modelling, including RS, proximal soil sensing (PSS), the use of AI (here, machine learning (ML) and deep learning (DL)), and biogeochemical modelling. We highlight applications, strengths and limitations of each approach. Section 7 of the review focuses on integration approaches, such as RS/sensor technology – model integration, biogeochemical model – AI – RS/sensor technology integration. In addition to providing an overview of current methodological and integrative work, this review aims to address the following questions: 1) what are the respective strengths and limitations of AI‐based and biogeochemical models for SOC prediction, and how do they perform under varying environmental conditions and data availability? 2) how can hybrid modelling approaches effectively integrate AI, process‐based models, and RS data to overcome current predictive limitations? 3) what are the major challenges, such as data scarcity, calibration complexity, and model interpretability, that hinder the broader application of integrated SOC prediction frameworks, and 4) how can current approaches be improved? Our overarching aims were to 1) uncover synergies and antagonisms between approaches and 2) ascertain opportunities for improving SOC prediction accuracy.

## 2 SOC Fundamentals

**Figure 1:**
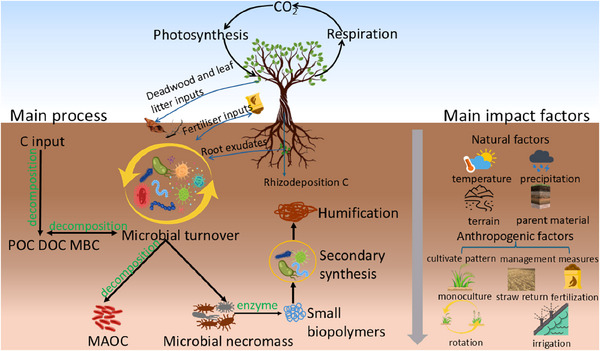
Key processes and influencing factors in soil organic carbon (SOC) formation. Abbreviations: particulate organic carbon (POC), dissolved organic carbon (DOC), microbial biomass carbon (MBC), and mineral associated organic carbon (MAOC).

Soil supports a diverse range of ecosystem services, including nutrient supply, maintenance of vegetation productivity, provision of biological habitats, organic carbon sequestration, and pollutant breakdown.^[^ ^11^ ^]^ Many of these functions rely on soil organic matter (SOM). SOC is the measurable carbon component and a major constituent of SOM, increasing SOM can suSOC sequestration. SOC sequestration sustain agroecosystem productivity but is also seen as an effective strategy for reducing atmospheric CO_2_.^[^ ^12^ ^]^ As shown in **Figure** **1**, primary ecosystem carbon inputs include animal and plant residues, rhizosphere sediments, microbial residues, and exogenous carbon input.^[^ ^13^ ^]^ Among these, microbial residual carbon contributes 51% of SOC, representing the majority of SOC input.^[^ ^14^ ^]^ The composition and transformation of ecosystem carbon inputs determine both the magnitude and persistence of soil carbon sinks.

Natural and anthropogenic factors drive changes in SOC stocks through perturbed carbon inputs, decomposition rates, and stabilization processes.^[^ ^15^ ^]^ Understanding and accurately monitoring these dynamics help informing agronomic practices, environmental sustainability, and climate mitigation strategies. For a detailed discussion of the effects of natural and anthropogenic factors on SOC dynamics, please refer to Text S1 (Supporting Information).

## 3 Pathways for Estimation SOC

**Figure 2:**
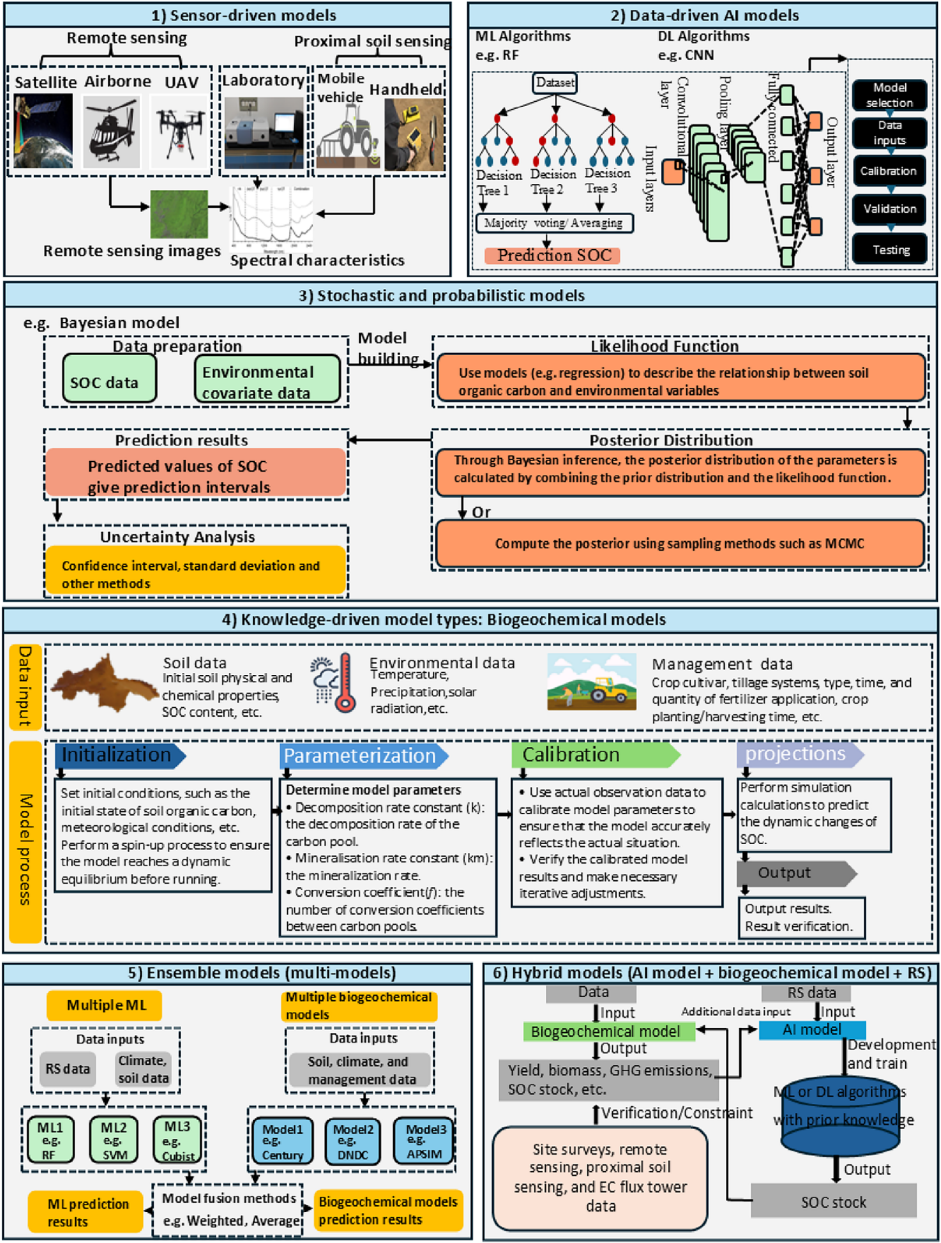
Model archetypes for quantifying soil organic carbon (SOC) stocks and fluxes. Abbreviations: unmanned aerial vehicle (UAV), artificial Intelligence (AI), machine Learning (ML), convolutional neural network (CNN), soil organic carbon (SOC), remote sensing (RS), denitrification‐decomposition model (DNDC), and agricultural production systems simulator (APSIM).

The balance between soil carbon inputs and outputs is a fundamental determinant of SOC sequestration and permanence. Soil management focusing on SOC requires not only an understanding of SOC stock distribution but also the ability to monitor and predict dynamic carbon fluxes, including processes such as carbon fixation, release, and transformation.^[^ ^16^ ^]^ Direct measurements and modelling approaches were used to estimate SOC stocks and fluxes in response to land management practices and climate change. Conventional soil core sampling methods are labor‐intensive, time‐consuming, and costly. While necessary, it also highlights the necessity of adopting approaches that can supplement SOC estimation, such as modelling. Modelling frameworks can support projections of SOC accumulation or depletion under different land‐use and management scenarios, as well as simulation of future changes in SOC.^[^ ^5a^ ^]^ As shown in **Figure** **2**, SOC modelling method can be categorized into six groups:

1. **Sensor‐driven models: RS and PSS informed modelling**

For large geographical areas soil sampling using physical cores is often impractical due to logistical and financial constraints. RS has emerged as a valuable tool for digital SOC mapping, particularly for studies with large scale (e.g., regional to global). RS provide proxies for SOC estimation by capturing soil surface spectral characteristics (e.g., absorption features in visible‐to‐shortwave infrared bands and derived indices).^[^ ^17^ ^]^ Most passive RS sensors, such as optical (e.g., Landsat, Sentinel‐2) and hyperspectral sensors (e.g., Hyperion), capture surface reflectance and vegetation indices, which are correlated with SOC content.^[^ ^18^ ^]^ The limitation with RS‐based approaches is that they are often constrained by cloud cover and better work bare soil for direct soil sensing. RS‐based approaches cannot account for SOC variation with depth. Integrating RS data with ground‐based observations can improve the sensor and model calibration and refine predictions across heterogeneous landscapes. For example, Bao et al.^[^ ^19^ ^]^ collected 324 surface soil samples and measured their laboratory Vis‐NIR spectra and SOC contents. They found that SOC prediction based on Sentinel‐2A data alone achieved an R^2^ of 0.57, while integrating Sentinel‐2A with laboratory Vis‐NIR spectra improved the prediction accuracy significantly (R^2^ = 0.78). (See Section 4.1 for detailed discussion).

PSS‐informed modelling uses laboratory‐ or field‐based sensors to infer soil properties, enabling near real‐time acquisition of SOC data. Most of current PSS are based on visible and near‐infrared spectroscopy. Spectral measurements are cost effective and can therefore support the quantification of SOC at different depths. For example, the Digital Soil Core (DSC) probe proposed by Grunwald et al.^[^ ^20^ ^]^ facilitates in‐situ soil profile characterization down to ≈1.2 m, which can support the spatial and temporal resolution of SOC measurements. Ground‐penetrating radar (GPR), another PSS, estimates SOC indirectly by analyzing the propagation characteristics of electromagnetic waves in the soil, such as wave velocity and signal amplitude. Since SOC has a relatively low dielectric constant, soils with higher SOC content tend to exhibit lower overall permittivity. By establishing empirical models that relate dielectric permittivity to SOC, while accounting for confounding factors such as soil moisture and texture, GPR enables non‐destructive estimation of subsurface SOC.^[^ ^21^ ^]^ (See Section 4.2 for detailed discussion).

2. **Data‐driven model types: AI models (ML and DL)**

ML techniques have become important in SOC modelling, as they enable the identification of complex relationships between SOC and environmental covariates in large datasets.^[^ ^22^ ^]^ These models are based on statistical learning to predict SOC concentrations, stocks, and fluxes based on observed patterns in the data, with applications extending to soil respiration and sequestration rate estimation.^[^ ^23^ ^]^ The quality of predictions made by ML is a function of the quality and quantity of training data, and the feature extraction. In addition, applying the model beyond its calibration range may lead to unreliable projections. (See Section 5 for detailed discussion).

DL, a subset of ML, use feature extraction from large, high‐dimensional datasets.^[^ ^24^ ^]^ As new data become available, DL models can be retrained using these data, thereby better capturing the influence of changing environmental and management conditions on SOC outcomes while maintaining predictive performance.^[^ ^25^ ^]^ (See Section 5 for detailed discussion). Nevertheless, DL models require large calibration datasets, and are usually long to calibrate, as well as require significant storage resources. They are also more prone to overfitting and require technical expertise for implementation.^[^ ^26^ ^]^

Both ML and DL are limited by dependence on data quality, limited interpretability, and scalability issues in real‐world SOC applications. (See Section 5.3 for detailed discussion).

3. **Stochastic and probabilistic models**

Stochastic approaches – such as Markov Chain Monte Carlo methods – can be used on deterministic models for error propagation, a step often conducted to determine uncertainty associated with model parameters or structure.^[^ ^27^ ^]^ Probabilistic techniques, such as Bayesian frameworks and Monte Carlo simulations, capture SOC variability by incorporating probabilistic distributions rather than relying solely on deterministic relationships.^[^ ^28^ ^]^ Pelletier et al.^[^ ^29^ ^]^ used a Bayesian‐based framework to calibrate SOC model parameters, which reduced the uncertainty in SOC prediction. While valuable for uncertainty quantification, these approaches face key challenges: 1) high computational costs, especially for complex models or large datasets; 2) sensitivity to prior distribution assumptions that may introduce bias if assumptions are weak; 3) stochastic models often perform poorly when applied to conditions that differ substantially from those present in the training data‐they are generally effective for interpolation but unreliable for extrapolation; and 4) complex interpretation of probabilistic outputs compared to deterministic results.^[^ ^30^ ^]^

4. **Knowledge‐driven models: biogeochemical models (mechanistic process‐based simulation models)**

Biogeochemical models simulate SOC dynamics using process‐based mathematical representations of carbon cycling within plant‐soil systems. These models typically consist of conceptual carbon pools that represent decomposition, stabilization, and transformation processes, allowing for the simulation of SOC changes under different environmental conditions and land‐use practices.^[^ ^31^ ^]^ Some biogeochemical models also integrate crop growth simulations, further enhancing their applicability in agricultural systems.^[^ ^32^ ^]^ These models are typically driven by climate data, such that outputs are a function of changes in weather data used as inputs. It is important to recognize that many crop simulation models were developed from limited plot‐based experiments and laboratory studies. As a result, they are often applied beyond their original physicochemical and biological domain ranges, which may compromise model performance and reliability. Therefore, users are strongly encouraged to evaluate the suitability of a given model for its intended application before deployment, particularly the aims with which that model was built, the calibration data zone, and the underpinning assumptions used to create the model structure. (See Section 6 for detailed discussion).

5. **Ensemble models (multi‐model approaches)**

Ensemble models integrate multiple models of the same type—either multiple AI algorithms or multiple biogeochemical models—to mitigate prediction errors and uncertainties.^[^ ^22^, ^33^ ^]^ However, variability associated with model ensembles can increase relative to individual models, and the process of calibrating and synthesizing multiple models requires significant capability (proficient model users); some authors have shown that model outputs depend as much or more on the model users than they do the model structure or parameterization.^[^ ^9^, ^34^ ^]^ (See Section 7.3 for detailed discussion).

6. **Hybrid models**

Hybrid models combine different types of models and data sources (e.g., ML/DL models, biogeochemical models, and RS data).^[^ ^35^ ^]^ By integrating mechanistic knowledge from biogeochemical models with predictive capabilities of data‐driven models, hybrid frameworks offer greater adaptability to diverse research contexts and environmental conditions. (See Section 7.4 for detailed discussion).

Methods employed for SOC simulation are diverse. Each method comes with its purpose and assumptions, and thus, its strengths and limitations. To provide modelers with an understanding of the application and selection of models, this review first explores the three main concepts used for SOC prediction, namely sensor‐driven models (**4. Sensing technologies for SOC prediction**), AI models (**5. Predicting SOC using AI**), and biogeochemical models (**6. Biogeochemical models for SOC simulation**). Second, the integration of RS, AI, and biogeochemical models is discussed in section 7.

## 4 Sensing Technologies for SOC Prediction

### 4.1 Global Trends and Technological Advances in SOC Prediction using RS

RS offers extensive, frequent, and multi‐dimensional spectral information, with developers aspiring to improve SOC monitoring via proxies for SOC drivers. RS is particularly useful for large‐scale areas (e.g., grasslands and pastures) where field sampling is challenging. RS data originate from multiple platforms, including unmanned aerial vehicles (UAVs), airborne sensors, and satellites.^[^ ^36^ ^]^ This section summarizes global trends in RS‐based SOC prediction, the commonly used RS data types, and practical applications.

**Figure 3:**
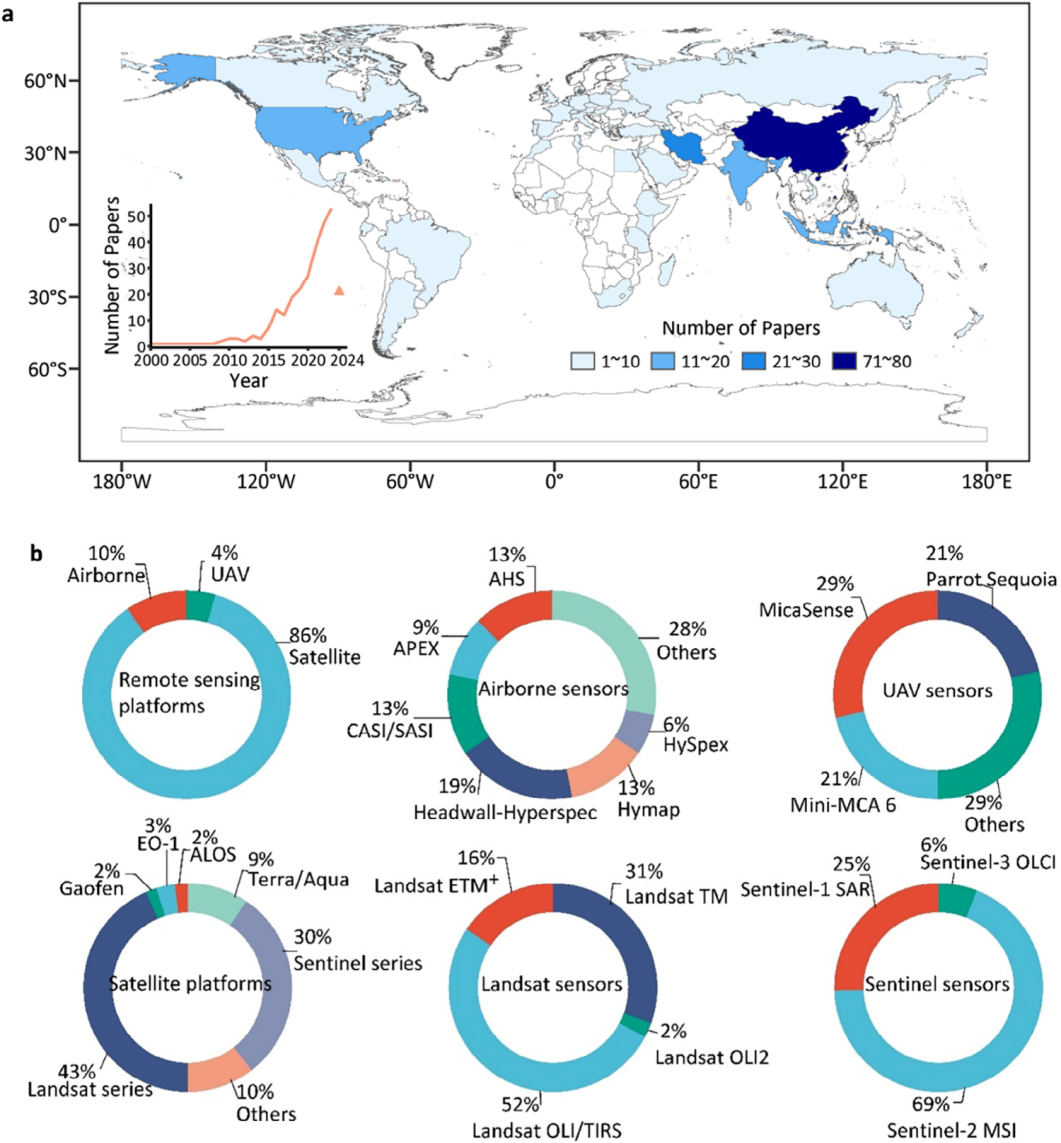
Global analysis of studies (total N = 279) employing remote sensing (RS) for soil organic carbon (SOC) prediction. a) Geographic distribution of papers published between January 2000 and June 2024 that utilize RS as a data source for predicting SOC. Inset chart highlights growth in number of publications over this period. b) Relative frequency of RS platforms and sensors used in the aforementioned studies, characterized as satellite (e.g., Operational Land Imager/Thermal Infrared Sensor (OLI/TIRS), Thematic Mapper (TM), and Synthetic Aperture Radar (SAR) sensors), airborne (e.g., Airborne Hyperspectral Sensor (AHS), Hyperspectral Imaging System (HySpex), and Headwall‐Hyperspec sensors) and unmanned aerial vehicle (UAV) (e.g., Parrot Sequoia and Mini‐MCA 6 sensors) platforms.

**Figure** **3**a illustrates the global distribution of RS‐based SOC studies, with most originating from the Northern Hemisphere, particularly China (80), Iran (28), and the United States (20). The number of related publications has increased since 2000, reaching 53 annually in 2023 (literature search methods see Text S3, Supporting Information). Figure 3b categorizes RS platforms and sensor utilization, showing that satellite imagery was used in 86% of studies, while airborne and UAV platforms accounted for 10% and 4%, respectively. The dominance of satellite‐based RS is largely due to its accessibility and capacity to cover vast areas, making it a valuable tool for SOC studies. Among satellite sensors, Landsat‐8′s Operational Land Imager and Thermal Infrared Sensor, as well as Sentinel‐2′s Multi‐Spectral Instrument are the most widely used. A brief review of these RS platforms and sensors is provided in Text S2 (Supplementary Information).

RS data can capture surface soil characteristics, serving as spatial references for SOC estimation. For instance, Wang et al.^[^ ^18^ ^]^ utilized Landsat‐8 spectral bands to derive nine environmental covariates, significantly improving SOC stock predictions for the 0–30 cm soil layer. Vegetation indices such as normalized difference vegetation index, enhanced vegetation index, and soil adjusted vegetation indexare key predictors of SOC variability. These indices reflect plant productivity, plant growth influences carbon inputs through root exudates and root residues.^[^ ^18^ ^]^ Airborne hyperspectral data (400–990 nm) effectively predict SOC with high spatial resolution.^[^ ^37^ ^]^ Within the 400–900 nm range, visible light bands are influenced by soil chromophores and humic acids, while near‐infrared bands correspond to functional group vibrations (e.g., C─H, C─O, N─H).^[^ ^38^ ^]^ UAV hyperspectral measurements require local calibration models and field sampling. Zhang et al.^[^ ^39^ ^]^ improved SOC prediction by integrating UAV spectral data with soil spectral libraries. UAVs using “structure from motion” can generate digital elevation models and terrain attributes, such as slope and topographic wetness index, commonly used in SOC modelling.

Despite its potential, RS‐based SOC estimation faces challenges. Soil heterogeneity, moisture, vegetation, and cloud cover distort spectral signals. Regional specificity limit broader applicability, requiring localized calibration and data fusion techniques. In addition, SOC stock estimates depend on auxiliary variables like bulk density, gravel content corrections, and are constrained by depth of sample and sampling frequency over time. Harmonizing and integrating global SOC data with multi‐source RS remains a significant opportunity for improving modelling efforts on SOC, such as model development, parameter uncertainty estimation, calibration, and validation.

### 4.2 Application of PSS Technology in SOC Prediction

PSS is commonly used for site‐specific estimation of SOC, particularly in environments where satellite‐based RS is constrained—for example, under dense vegetation cover, where cloud cover is frequent and/or at fine spatial scales.^[^ ^40^ ^]^ PSS involves deploying sensors within 2 meters of the soil material to obtain a spectrum which can then be correlated to SOC content and carbon fractions.^[^ ^41^ ^]^ Among these, diffuse reflectance spectroscopy is a key method that can be conducted both in the laboratory and in the field.^[^ ^42^ ^]^ PSS technology relies on the interaction of electromagnetic energy with soil components, with visible and infrared regions proving particularly effective. Common PSS techniques include visible‐near infrared (Vis‐NIR) and mid‐infrared spectroscopy, laser‐induced breakdown spectroscopy, and X‐ray fluorescence spectroscopy, which infer SOC content through calibration with a spectral library.^[^ ^40b^ ^]^ Laboratory‐based PSS is widely accepted due to controlled conditions, where factors like soil moisture and environmental conditions can be controlled.^[^ ^42b^ ^]^ Field‐based SOC estimation can be corrected for soil moisture effects through spectra preprocessing techniques such as orthogonal signal correction.^[^ ^42b^ ^]^

The field sensor can be mounted on a mobile vehicle (e.g., Veris's CoreScan or Landscan's Digital Soil Corer) or as a handheld instrument. For instance, Rodionov et al.^[^ ^43^ ^]^ mounted a Vis‐NIR spectrometer on a tractor, comparing stop‐and‐go with on‐the‐go data collection. Stop‐and‐go achieved promising SOC predictions (R^2^ = 0.65), though with lower accuracy than laboratory‐based models (R^2^ = 0.94). Handheld instruments, such as the ASD Field Spec III and Agilent 4300 handheld FTIR, have also been tested. Huteng et al.^[^ ^44^ ^]^ found that portable MIR spectroscopy performed comparably to benchtop instruments (R^2^ = 0.77–0.78 vs 0.73–0.85). Similarly, Cambou et al.^[^ ^45^ ^]^ reported SOC predictions using the Vis‐NIR ASC LabSpec 2500 with R^2^ values between 0.52–0.86 in the field and 0.68–0.76 across three different sites in France. Grunwald et al.^[^ ^20^ ^]^ highlight developments in multi‐sensor approaches. Viscarra Rossel et al.^[^ ^40a^ ^]^ introduced the Soil Condition Analysis System (SCANS), which combines Vis‐NIR spectroscopy, AGA for bulk density estimation, and digital imaging. SCANS supports SOC estimation in both lab and field applications, offering cost‐effective and flexible carbon accounting solutions.

### 4.3 Integrating Sensing Technologies for SOC Quantification

**Figure 4:**
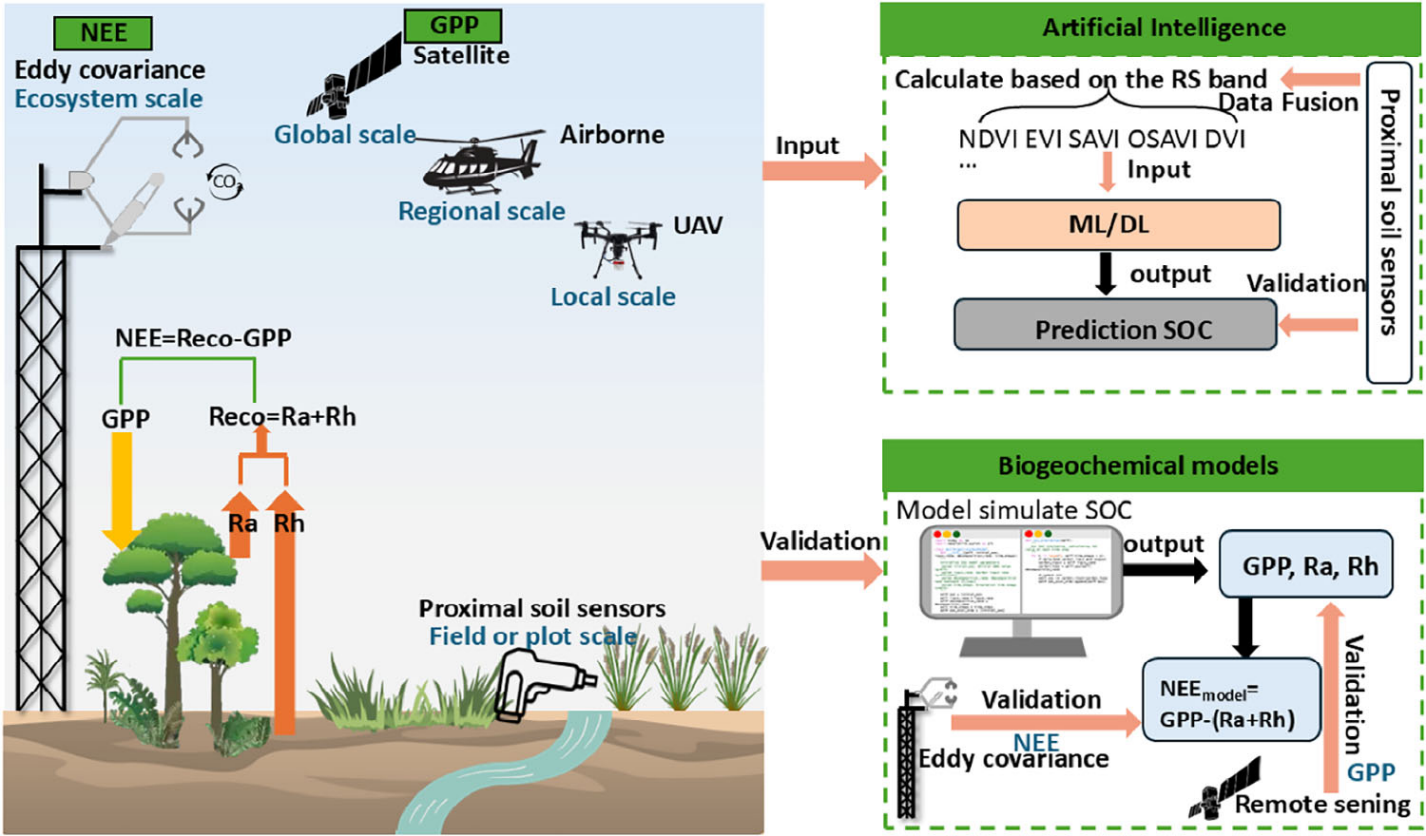
Conceptual workflow for quantifying and validating soil organic carbon (SOC) using remote sensing (RS), proximal soil sensing (PSS) and eddy covariance (EC) flux towers. Left panels depict various sensing technologies. Right panels demonstrate how these sensing technologies contribute to SOC prediction, showing their roles in data assimilation and model verification. The RS section includes satellite, airborne, and unmanned aerial vehicle (UAV)‐based spectral feature extraction and calculation of covariates such as NDVI (normalized difference vegetation index), EVI (enhanced vegetation index), SAVI (soil adjusted vegetation index), OSAVI (optimized soil adjusted vegetation index), and DVI (difference vegetation index) which are common vegetation indices that are used as proxies in the estimation of the SOC. PSS collect data at the field or plot scale, while the EC tower measures net ecosystem exchange (NEE) to validate the model. Abbreviations: autotrophic respiration (Ra), heterotrophic respiration (Rh), total ecosystem respiration (Reco), gross primary productivity (GPP).

As shown in **Figure** **4**, RS provides spectral information over broad areas, while PSS offers detailed local spectral information. Integrating RS and PSS can leverage the strengths of both, enabling fine‐scale SOC predictions.^[^ ^46^ ^]^ For example, Bao et al.^[^ ^19^ ^]^ integrated satellite observations with PSS data and applied a spectral reconstruction method to generate high spectral resolution imagery. They found that the reconstructed image improved the accuracy of SOC prediction (R^2^ = 0.78) compared with using only PSS data (R^2^ = 0.69) or RS data (R^2^ = 0.57). The method first used a soil type map to identify soil textures within the RS image then established a relationship between reflectance spectra of the image and PSS spectral data of sampling points from corresponding soil categories. A spectral response function was then employed to reconstruct the high spectral resolution image. Peng et al.^[^ ^47^ ^]^ similarly showed that fusing PSS, RS, and environmental geodata improved SOC prediction performance, with R^2^ increasing by ≈12–43% compared with using RS and PSS with environmental geodata separately. This integration compensates for limitations of individual sensors in spectral, spatial, and radiometric resolution, enabling generation of more contiguous SOC solution spaces while reducing noise and inconsistencies in the data. In addition, PSS data serve as ground‐truth references for calibrating and validating SOC estimates derived from RS.

Changes in ecosystem carbon stocks are determined by the mass balance between carbon input and output fluxes.^[^ ^48^ ^]^ Change in SOC over time (i.e., SOC flux) can be quantified using a carbon mass balance equation: ΔSOC = ‐net ecosystem carbon exchange (NEE) – annual yield.^[^ ^48^ ^]^ Eddy covariance (EC) flux towers provide continuous, direct NEE measurements at the ecosystem scale, offering high‐frequency carbon flux data.^[^ ^42a^ ^]^ In contrast, RS technology provides large‐scale, multi‐temporal information independent of field measurements, making it useful for extending EC flux observations over larger areas and longer periods.^[^ ^27^, ^49^ ^]^ AI models using ML algorithms performed well in predicting soil respiration (Rs), achieving an R^2^ of 0.89 and 0.86 for Rh. At the global scale, Rs was estimated at 85.5 Pg C yr^−1^, and Rh at 50.3 Pg C yr^−1.^ In contrast, 10 mechanistic models showed wide variability in results, ranging from 61.4 to 91.7 Pg C yr^−1^ (Rs) and 39.8 to 61.7 Pg C yr^−1^ (Rh).^[^ ^50^ ^]^ The global study by Huang et al.^[^ ^51^ ^]^ applied ML (random forest (RF) and support vector machines (SVM)) and DL (artificial neural networks (ANN)) to Rs modelling, achieving R^2^ values from 0.47 to 0.68 and RMSE between 148 and 429 g C m^−2^ yr^−1^. Boreal, temperate, and tropical regions contributed 15, 24, and 61%, respectively, to total mean annual global Rs. AI models explained 62–84% of interannual and inter‐site variabilities in annual Rs globally. RS data can compensate for EC's limited spatial coverage, while EC measurements provide high‐precision data for calibrating RS‐based carbon flux estimates. By integrating both sources into carbon assimilation models, regional and global carbon balances may be estimated more accurately than using either RS or EC data alone.^[^ ^52^ ^]^

A key requirement for successfully integrating multi‐source sensors is ensuring high spatial resolution and temporal overlap between field measurements.^[^ ^53^ ^]^ Researchers have integrated RS and PSS data through consistent aggregation methods to address this challenge. For example, Wang et al.^[^ ^54^ ^]^ improved SOC prediction accuracy by pre‐selecting spectral bands with proximal spectrometers that matched those of RS images, extracting a portion of bands for analysis. Temporally, time‐series data can be harmonized using interpolation or feature extraction within defined temporal windows (e.g., monthly or seasonal composites). An emerging trend in SOC modelling is the development of space‐time SOC models using RS datasets structured as data cubes.^[^ ^8b^ ^]^ This approach uses multiple points in space to compensate for taking measurements over time (because measuring SOC over time can take decades to realize significant changes). Additionally, fusion techniques, such as DL models that can accommodate heterogeneous data structures, offer another solution for handling complex spatiotemporal dependencies.

## 5 Predicting SOC using AI

### 5.1 Advancing SOC estimation with AI

**Figure 5:**
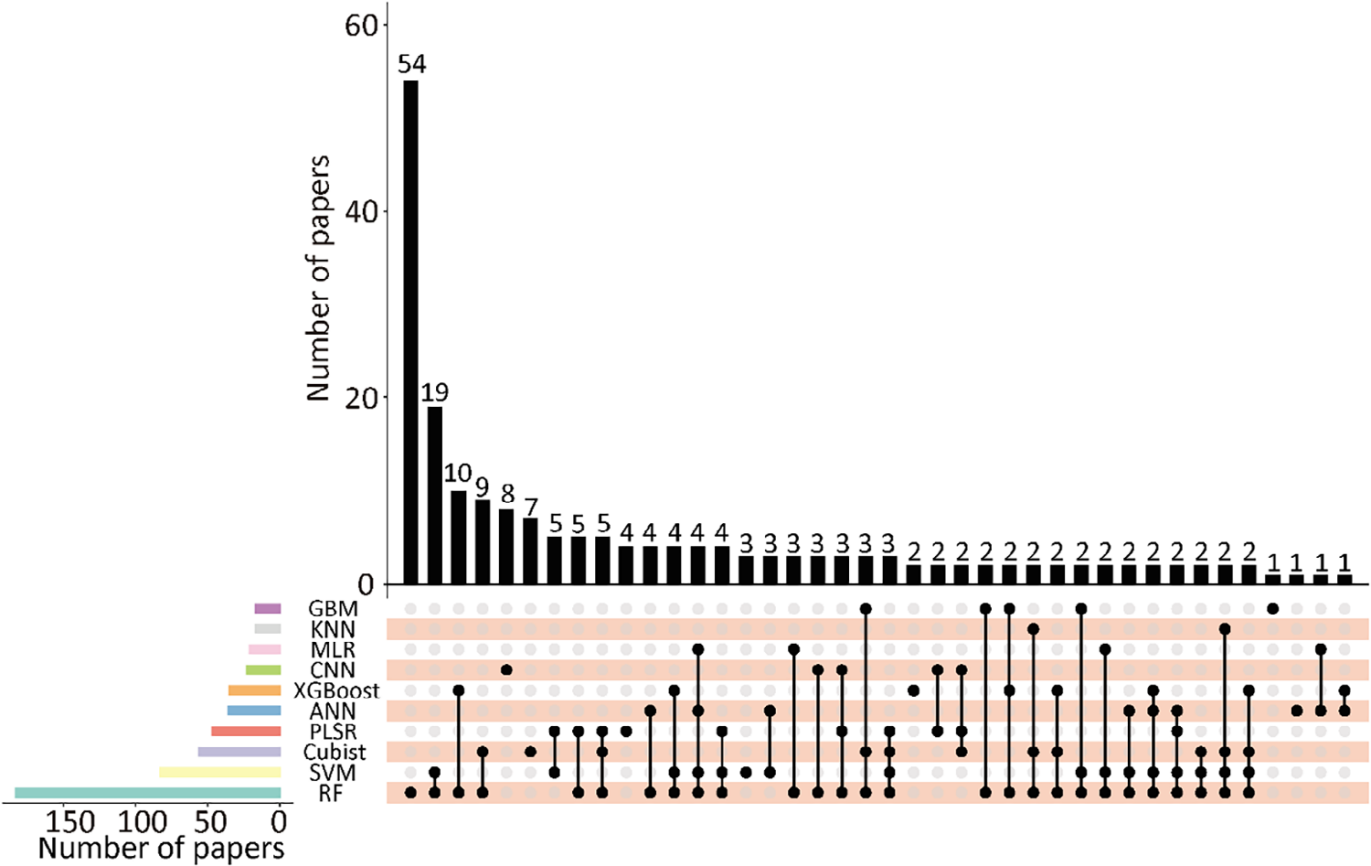
Publications used AI for soil organic carbon (SOC) prediction. Bar chart on the left shows total publications for each algorithm. The dot chart below illustrates intersections between algorithms, with connecting lines indicating instances where two or more algorithms are applied together in a single study. Top bar chart displays number of publications associated with these algorithmic intersections. Abbreviations: Random Forest (RF), Support Vector Machine (SVM), Partial Least Squares Regression (PLSR), Artificial Neural Networks (ANN), Extreme Gradient Boosting (XGBoost), convolutional neural networks (CNN), Multiple Linear Regression (MLR), k‐Nearest Neighbor (KNN), and Gradient Boosting Machine (GBM). Data sourced from Web of Science 12 December 2024.

AI predicts SOC spatial distribution by relating point‐based SOC observations to environmental covariates such as topography, climate, and remote sensing data. Our review identified the top 10 AI algorithms used for SOC prediction, based on publication volume, as shown in **Figure** **5** (literature search methods see Text S4, Supporting Information). RF was the most frequently used, invoked in 182 studies, including those that combined RF with other algorithms. SVM and Cubist (a rule‐based ensemble regression model) followed in popularity. Among DL techniques, ANN, and convolutional neural networks (CNN) were the most commonly employed.

**Figure 6:**
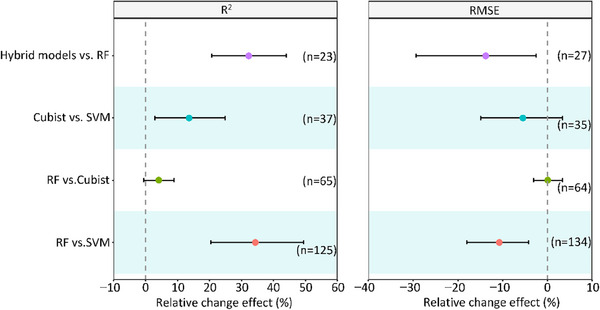
Comparison of the relative change effects in model performance for soil organic carbon (SOC) stock prediction between machine learning (ML) algorithms and hybrid models. Relative change is expressed as percentages for R^2^ (left panel) and RMSE (right panel). Comparisons are relative to the model listed on the right side of each pairing (e.g., “RF versus SVM” indicates that Random Forest (RF) is compared against Support Vector Machine (SVM)). Hybrid models refer to a combination of ML algorithms and other modelling approaches, including ensembles of ML techniques, geostatistical models (e.g., kriging), and biogeochemical models. Positive values indicate improvements in R^2^ or reductions in RMSE for the model listed on the left side of the comparison. Error bars represent 95% confidence intervals, with the number of studies included in each comparison provided in parentheses.

To assess algorithm performance, we conducted a meta‐analysis of the three most commonly used models, as shown in **Figure** **6**. Studies predominantly employed cross‐validation techniques such as leave‐one‐out or k‐fold validation, using the coefficient of determination (R^2^) to assess model fit and root mean square error (RMSE) to evaluate predictive accuracy. Our findings indicate that RF improves R^2^ by 34% and 4% compared to SVM and Cubist, respectively. Additionally, Cubist outperforms SVM, increasing R^2^ by 13%. Both RF and Cubist demonstrate superior accuracy, with higher R^2^ and lower RMSE values compared to other AI methods. The hybrid models in our meta‐analysis include ML ensembles, ML‐geostatistical integrations (e.g., regression kriging), and ML‐biogeochemical model combinations. As shown in Figure 6, hybrid models often surpass individual models in predictive accuracy.^[^ ^22^ ^]^ This will be discussed in detail in Section 7.

Despite these insights, several limitations must be considered. A major challenge lies in the comparability of R^2^ and RMSE across studies, as variations in SOC dataset heterogeneity significantly influence these metrics. Studies conducted over smaller, more homogenous areas tend to report higher R^2^ and lower RMSE than those covering larger, more diverse regions, introducing potential biases in cross‐study comparisons. Furthermore, inconsistencies in R^2^ calculation methodologies hinder direct comparisons, complicating meta‐analyses. These challenges highlight the need for standardized reporting practices and consistent evaluation metrics in SOC research. Establishing such standards would enhance the reliability and comparability of findings, facilitating cross‐study analyses and improving AI‐driven SOC prediction accuracy.

### 5.2 ML for SOC Prediction

ML, a subset of AI, identifies patterns in datasets to make predictions.^[^ ^22^ ^]^ Unlike parametric models, ML did not rely on strict assumptions about data distribution. This made it well‐suited for capturing the complex, nonlinear relationships often observed in environmental data.^[^ ^6b^ ^]^ Studies suggested that RF and Cubist better captured the nonlinear relationships between predictive variables and SOC, leading to higher prediction accuracy than SVM.^[^ ^55^ ^]^ Keskin et al.^[^ ^56^ ^]^ compared seven ML models and one geostatistical method (ordinary kriging) for modelling SOC stocks and carbon fractions in Florida, USA, and found RF to be the most effective. However, results varied. Shafizadeh‐Moghadam et al.^[^ ^57^ ^]^ reported that SVM outperformed RF in SOC content prediction, likely due to a smaller training dataset. Were et al.^[^ ^58^ ^]^ found SVM to be the best predictor of landscape SOC stocks, possibly due to differences in study area, topography, sampling density, or auxiliary data quality. A recent study applied RF, SVM, XGBoost, and DNN algorithms to predict SOC under different land use types and found that XGBoost achieved the best performance (R^2^ = 0.73) when soil sample size was limited and computational efficiency was a concern.^[^ ^59^ ^]^ This superiority was attributed to its ability to handle heterogeneous data, capture complex relationships, and offer flexible optimization. These findings highlight that no single ML algorithm is universally superior, as the prediction accuracy of SOC models is highly context‐dependent. Specifically, spatial modelling performance is influenced by the sensitivity of each learning model to local geographic features, the size and quality of the input data, and the interactions among environmental covariates.^[^ ^59^ ^]^ This underscores the need for high‐quality prediction data and careful model calibration tailored to specific case studies.

### 5.3 DL for SOC Prediction

With advancements in AI, ML has evolved into many subset classifications, including DL, which has shown significant potential in processing complex soil spectral data.^[^ ^24b^ ^]^ An advantage of DL is its ability to extract features for classification through multiple layers of adaptive computational units (e.g., hidden nodes and layers), coupled to algorithms to model input‐output relationships.^[^ ^24a^ ^]^ Although our meta‐analysis did not directly compare DL and ML methods, previous studies suggested that DL generally outperforms traditional ML in SOC prediction. For instance, Hong et al.^[^ ^60^ ^]^ found that CNN outperformed PLSR and Cubist in full‐spectral SOC modelling. Similarly, Padarian et al.^[^ ^61^ ^]^ showed that multi‐task CNN reduced error by 87% compared to PLSR and by 62% compared to Cubist when predicting SOC content from spectral data. The capacity of DL to extract features makes it more advantageous when processing high‐dimensional data (such as spectral data) compared to parametric models.^[^ ^61^ ^]^ However, previous studies also acknowledge that DL models have deep and complex architectures, which typically require large amounts of training data. In the context of SOC prediction, RS data were often limited by cloud cover and the scarcity of bare soil observations, resulting in small sample sizes that may affect model performance. To address this, Yuan et al.^[^ ^62^ ^]^ proposed pre‐training, fine‐tuning, and domain adaptation in transfer learning, which are helpful for solving the small sample problem in environmental remote sensing. Beyond data limitations, DL models struggle to reveal functional relationships between spectral information and soil properties, which may hinder our understanding of key predictive factors.^[^ ^63^ ^]^ In addition, DL is usually accompanied by higher computational costs and training time.^[^ ^58^ ^]^

### 5.4 ML Versus DL: Method Selection under Different Conditions

In selecting an appropriate model for SOC prediction, understanding the differences between ML and DL is crucial. Based on **Table** **1**, the choice of method depends on several factors, including the amount of available data, the complexity of the problem, and the computational resources at hand.

**Table 1:** Comparison of the advantages and disadvantages of machine learning and deep learning in SOC prediction, including performance features and preferable applications.

Comparison Dimension	Machine learning	Deep learning	Refs.
**Training data requirement**	Performs better with large datasets	Typically requires very large amounts of training data	[61]
**Computational resource requirements**	Low, can run on standard computers	High, requires GPU or high‐performance computing resources	[64]
**Model structure**	Shallow Architecture (1‐2 functional layers), or tree‐based structure	Deep Network Architecture (5‐100+ hidden layers)	[62]
**Feature extraction**	Requires manual feature engineering	Automatically extracts features	[24]
**Preferred applications**	Lab/field point‐scale prediction Small‐medium regional modelling Scenarios requiring model interpretability	Multi‐source remote sensing data fusion High‐dimensional spectral processing Spatiotemporal sequence modelling	[65]

### 5.5 The Challenges of AI Techniques

Despite the clear advantages of AI algorithms, three key challenges remain in current research.^[^ ^66^ ^]^

1. **Data availability and quality**

High‐quality SOC measurements are sparse or unevenly distributed, particularly across space and time, limiting model generalization.^[^ ^24^, ^35^ ^]^ Accurate SOC stock prediction requires intensive sampling efforts.^[^ ^8^, ^67^ ^]^ The calculation of SOC stocks is limited by the lack of data on bulk density and gravel content. While AI methods are flexible, their effectiveness hinges on careful sample selection and parameter tuning to avoid overfitting.

2. **Lack of interpretability (“black box” Issue)**

AI models often lack transparency in linking input variables to SOC outcomes. To address this, interpretability tools such as Shapley values, permutation importance, and partial dependence plots are used to quantify variable contributions. Feature selection methods like Boruta also help identify key predictors.^[^ ^66^, ^68^ ^]^

3. **Limited extrapolation capabilities**

Most AI models are trained on historical SOC data and struggle to forecast temporal changes under evolving land use or climate scenarios.^[^ ^24a^ ^]^ In contrast, process‐based models incorporate mechanistic understanding of SOC cycling, enabling better prediction of future trends, or application to a different environmental context.^[^ ^8b^ ^]^

## 6 Biogeochemical Models for SOC Simulation

### 6.1 Trends in SOC Biogeochemical Modelling

**Figure 7:**
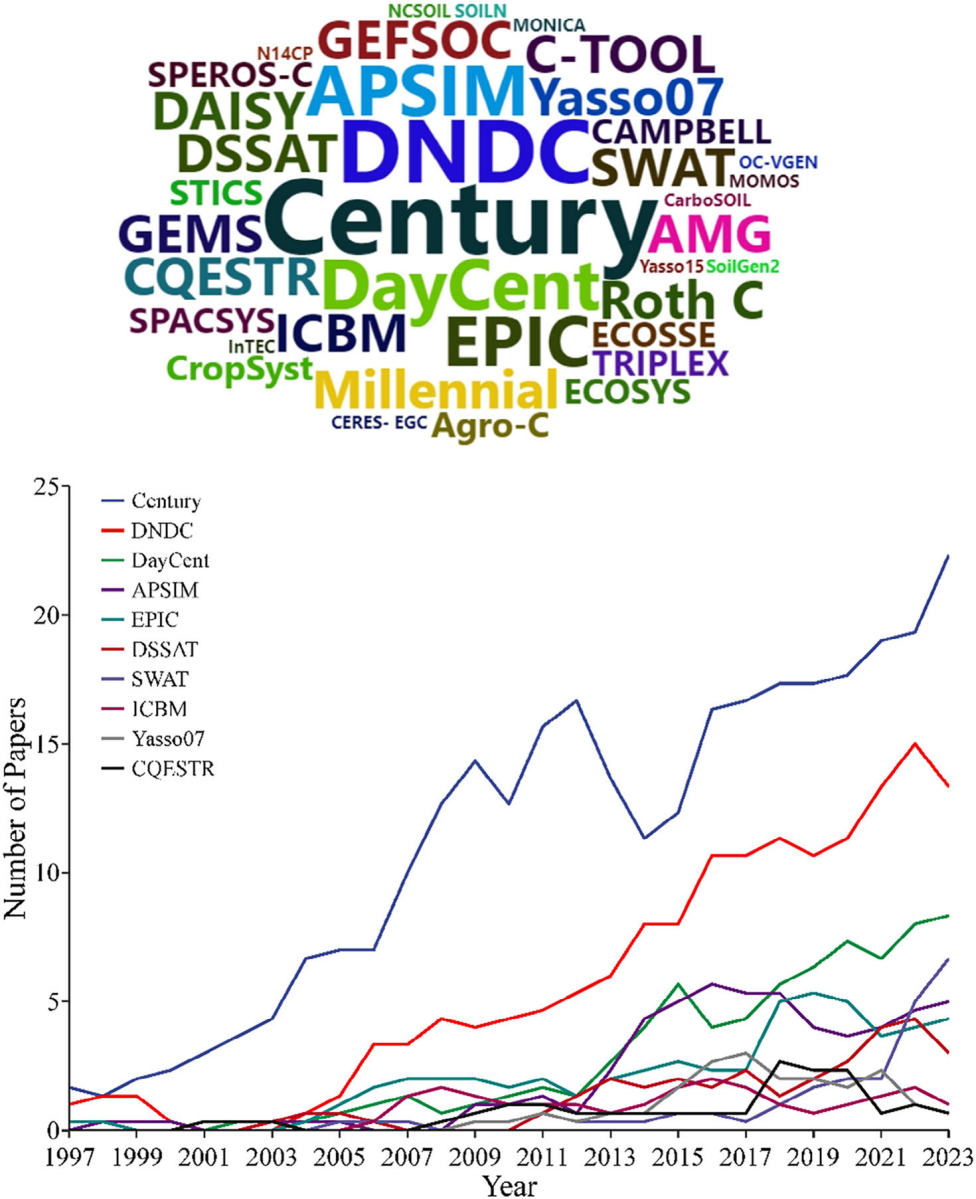
Word cloud and publication volume analysis of biogeochemical soil organic carbon (SOC) models. The upper panel presents a word cloud where larger words indicate higher frequency in the literature. The lower panel shows the publication volume for the top 10 models highlighted in the word cloud. Data sourced from the Web of Science, accessed on January 16, 2024.

Biogeochemical models are often preferred for SOC monitoring or temporal prediction because they accurately describe longitudinal effects of SOC inputs and loss as a function of climate.^[^ ^69^ ^]^ To understand the models applied to SOC prediction, we conducted a literature review (literature search methods see Text S5, Supporting Information). As shown in **Figure** **7**, we revealed a clear upward trend in SOC simulation research over the past two decades. This trend may be attributed to the continuous development and optimization of models, the gradual improvement in the accuracy of simulation results, and the significant advancements in data acquisition and sharing. Additionally, with government policy support and the growing emphasis on the carbon market by industries, the expansion of the carbon market has also provided a strong impetus for SOC simulation research.

**Figure 8:**
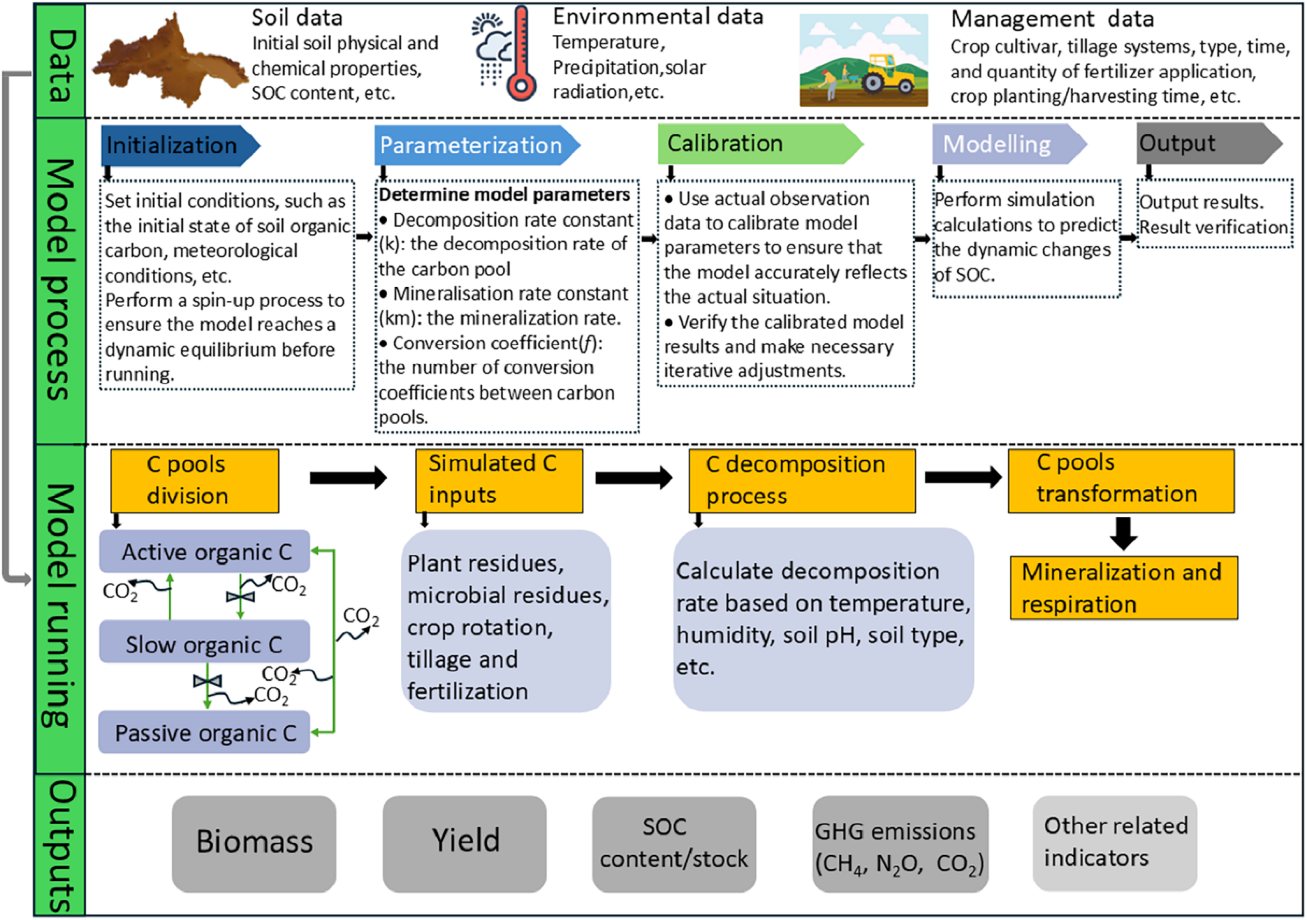
Workflow of a biogeochemical model simulating soil organic carbon (SOC) dynamics using CENTURY as an example.

Biogeochemical models incorporate processes such as water and nutrient inputs, carbon allocation, crop yield, and litter production to simulate crop growth and SOC decomposition processes.^[^ ^32a^ ^]^ While the mathematical foundations of biogeochemical and AI models are fundamentally different, their application workflows often share similarities. Common steps include selecting measured SOC, meteorological, and soil data, followed by model spin‐off, initialization, calibration, validation, project modelling, and measurement. **Figure** **8** illustrates the basic workflow of a typical biogeochemical model.

In SOC stock prediction, a critical initial step is determining the size of SOC pools. These pools are generally categorized into three types: active/labile SOC, intermediate/humic SOC, and passive/inert/recalcitrant SOC.^[^ ^70^ ^]^ However, significant debate exists in how users should conceptualize and partition these pools.^[^ ^34^, ^71^ ^]^ Here, we categorize workflows into two types (with and without spin‐up).^[^ ^31^ ^]^ “Spin‐up” is also referred to as “model equilibration”, i.e., the period before the project period that allows modelled SOC pools to stabilize over time. For long‐term SOC simulations, modelers often assume that the SOC pools are in equilibrium or “steady state” before implementing experimental treatments or management changes.^[^ ^31^, ^32^ ^]^ In practice, many soils may not have stable SOC. Spin‐up is commonly used as proxies to simulate “steady state” conditions by running models for long periods, e.g., Century allows users to spin‐up the model for hundreds of years prior to the analytical phase. Some authors have replaced simulated conceptual pools with measured SOC fractions in the aim of enabling more accurate initialization.^[^ ^72^ ^]^ Other users follow a long period of spin‐up with a shorter “burn‐in” phase, which tends to use more recent RS data to calibrate above‐ground biomass production, although this approach perturbs simulated SOC stability prior to the analytical phase. Following the spin‐up and burn‐in phases, measured SOC (often obtained in the field by coring) is used to initialize SOC pools as well as other variables, including management, surface residues, soil moisture, and mineral soil nitrogen.

Calibration involves adjusting model parameters to ensure accurate simulation of observed SOC data either using manual or automated approaches.^[^ ^73^ ^]^ For SOC, it is crucial to ensure transparency in data limitations and model calibration procedures. A group of data independent from the calibration dataset must be used to evaluate model performance once the model has been calibrated. The model validation process involves comparing simulations from the calibrated model against an independent set of measurements (i.e., not used for calibration). Validation thus captures all forms of uncertainty, including field and laboratory sampling, model initialization, parameterization, validation and structure (the equations used). When new measurements become available, previous calibrations can be repeated with the new data, a process some call model “true‐up”.

### 6.2 Pros and Cons Associated with Common Biogeochemical SOC Models

**Figure 9:**
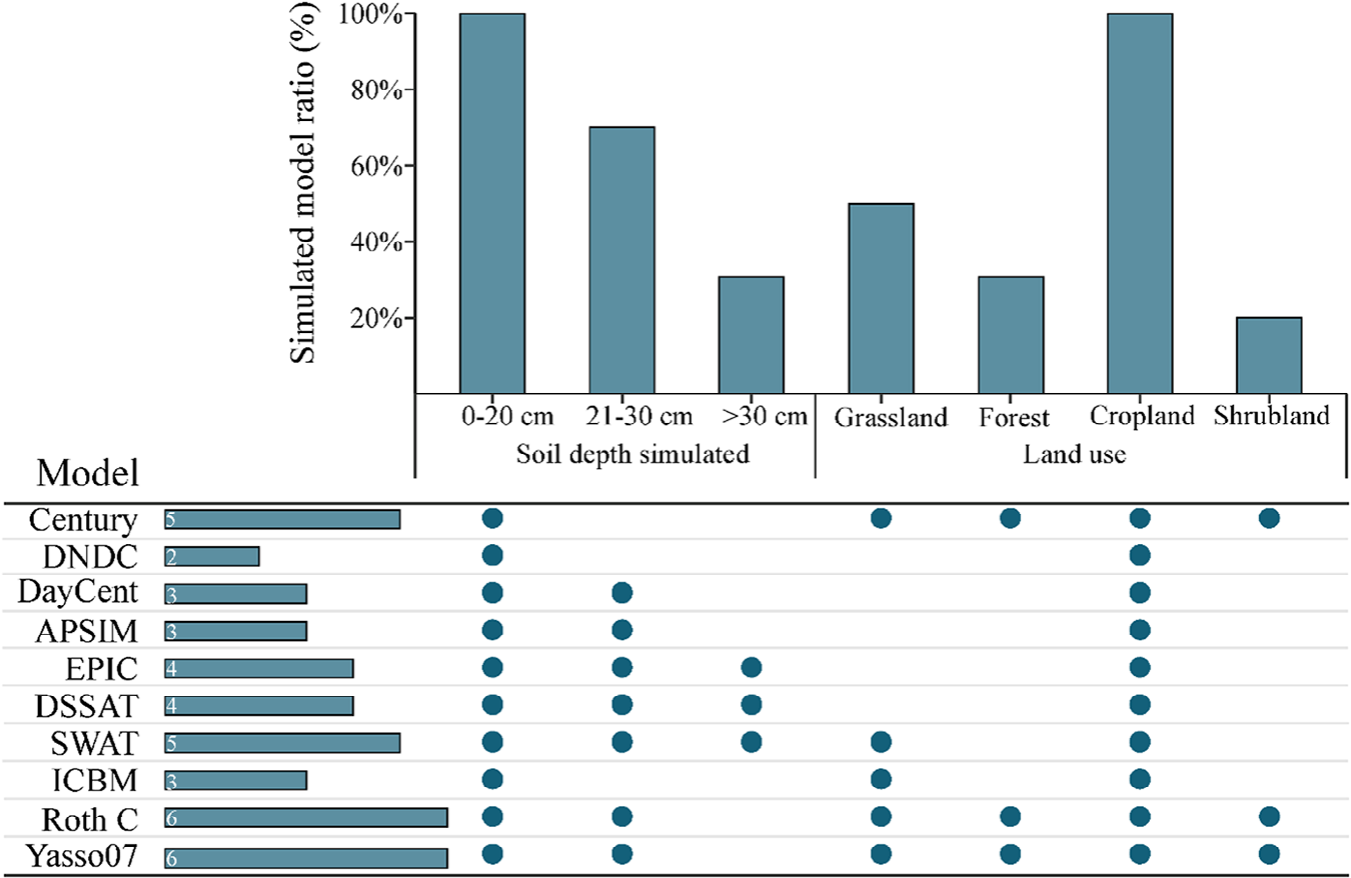
Overview of ten contemporary biogeochemical models, including capabilities for simulating soil organic carbon (SOC) under different depths and land use. Top panel shows percentage of models that simulate SOC at various soil depths and land uses. Bottom panel compares model coverages, with blue circles representing simulation capability.

As shown in **Figure** **9**, Most models are capable of simulating SOC in the top 20 cm of soil, but given that many soils are deeper than this arbitrary threshold, insights into SOC fluxes deeper than 20 cm may be obscured. Although the mineralization rate of soil carbon shows a decreasing trend in deeper soils, the decomposition rate does not always decrease with increasing soil depth.^[^ ^74^ ^]^ Physical disturbances such as crop root growth, animal activity, and human cultivation can transport surface organic matter to deeper layers, potentially increasing the organic carbon decomposition rate in deeper soils.^[^ ^75^ ^]^ These observations suggest that when selecting a model, it is crucial to consider the impact of soil depth on the mineralization rate of organic carbon, microbial community structure, and environmental conditions to achieve more accurate results. We found that most SOC models are primarily designed for croplands, forests, and/or grasslands, with fewer models, such as Century and Yasso07, being validated for various land use.

**Table** **2** compares parameters required for different SOC models. Soil parameters to be initialized typically include soil physico‐chemical properties. Most models are driven by climatic variables, such as solar radiation, relative humidity, temperature, and wind speed, and require users to input management factors such as crop type, irrigation, fertilizer (type, timing, and quantity), and crop planting/harvesting schedules. Most models function on a monthly (e.g., Century) or daily time step (e.g., DayCent), while in some instances, sub‐daily time steps are used. Yasso07 operates with an annual time step with highly aggregated processes in the model structure. Models with shorter time steps typically require more extensive input data of environmental and management factors, enabling them to respond more accurately to seasonal variations and management changes in SOC dynamics.^[^ ^76^ ^]^ In contrast, the inventory model Yasso07 is insufficient to represent ecosystem processes that occur at fine time scale (minutes to days to months), but it is considered acceptable for simulating annual SOC changes in forest ecosystems, as it can operate with relatively limited input data.^[^ ^77^ ^]^ However, it may not have the necessary temporal fidelity to effectively capture the direct impacts of extreme weather events or sudden changes in management practices. It is crucial to note that gathering more observational data significantly enhances the rigorous validation of all model outputs.^[^ ^78^ ^]^

**Table 2:** Advantages and disadvantages of biogeochemical models for simulating SOC (based on 10 frequently used models presented in Figure 7).

Models	Version	Carbon pools	Model inputs			Disadvantages	Refs.
			Soil parameters	Climatic drivers	Management information requirements		
Century^[^ ^86^ ^]^	4.0	5‐7^[^ ^33^, ^86^ ^]^	pH, soil texture (sand, silt and Clay), bulk density, fraction of excess water lost by drainage, initial values for wilting point, N deposition rate	Monthly total precipitation, maximum/minimum average temperature	Crop cultivar, tillage systems, type, time, and quantity of fertilizer application, crop planting/harvesting time, etc	Simulated rigour of forest ecosystems is not as good as that of farmland and grassland ecosystems, and there is a certain degree of regional variability.	[87]
DNDC^[^ ^88^ ^]^	CAN	6^[^ ^31^, ^89^ ^]^	Clay content, organic carbon, bulk density, pH	Daily total precipitation, maximum/minimum temperature	Crop residue management, mineral fertilization inputs, manure carbon inputs, tillage systems, crop planting/harvesting time	Plant growth processes are simplified and thus difficult to match phenology, biomass. If the simulated biomass is inaccurate, there will be an intrinsic error in the subsequent SOC dynamics.	[69a]
DayCent^[^ ^90^ ^]^	4.5	5^[^ ^91^ ^]^	Organic carbon, organic N, bulk density, pH, soil texture, maximum/minimum soil temperature, soil hydraulic properties	Daily total precipitation, maximum/minimum temperature	Crop cultivar, tillage systems, fertilization management, crop planting/harvesting time, etc	Efforts to simulate the impact of crop cover on crop yield and SOC dynamics are very limited, as it is unable to capture annual changes in crop biomass.	[92]
APSIM^[^ ^93^ ^]^	7.10	3^[^ ^31^ ^]^	Bulk density, saturated water content, field capacity, wilting point, pH, organic carbon and initial mineral N	Daily total precipitation, maximum/minimum temperature, solar radiation	Tillage systems, type, time, and quantity of fertilizer application, crop planting/harvesting time	Does not accurately consider the impact of tillage on SOC decomposition, which may underestimate the rate of carbon decomposition and accelerate soil carbon loss. APSIM model was originally developed primarily based on clay soils, and its default parameters may implicitly assume a certain degree of physical protection for organic matter.	[94]
EPIC^[^ ^95^ ^]^	v3060	5^[^ ^96^ ^]^	Soil bulk density, soil water content at field capacity, saturated hydraulic conductivity, horizon thickness, topsoil clay content, soil texture, pH, cation exchange capacity, alkali‐hydrolyzable N, total N, P, and K content, and available P and K	Daily total precipitation, maximum/minimum temperature, solar radiation, relative humidity, and wind speed	Tillage systems, type, time, and quantity of fertilizer application, crop planting/harvesting time, and associated operation dates and quantities	Responsiveness to water stress could be improved. Highly sensitive to changes in soil type, soil texture, and other soil characteristics, which may result in differences in the model's predictions across different soils.	[97]
DSSAT^[^ ^98^ ^]^	v4.7	5^[^ ^31^ ^]^	Soil bulk density, texture, organic carbon, soil hydraulic parameters, wilting point, field capacity, saturated water content, soil texture, total N, pH	Daily total precipitation, maximum/minimum temperature, solar radiation	Crop cultivar, tillage systems, plant density, type, fertilization management, crop planting/harvesting time	Cannot simulate factors that affect SOC (such as temperature and microorganisms).	[99]
SWAT^[^ ^96^ ^]^	SWAT‐C	5^[^ ^100^ ^]^	Soil layer depth, soil texture, bulk density, organic carbon content, and soil erosion coefficient	Daily total precipitation, maximum/minimum temperature, solar radiation, relative humidity, and wind speed.	Crop planting/harvesting time, fertilization management, and irrigation applications	Cannot fully capture management factors that affect SOC, including soil aeration, tillage management factors, and interactions therein.	[101]
ICBM^[^ ^102^ ^]^	/2	2^[^ ^31^ ^]^	Content of sand, silt and clay	Daily total precipitation, maximum/minimum average temperature, humidity, cloudiness, and wind speed	Carbon inputs and crop yield	Sensitive to the initial SOC inventory. If the initial SOC inventory is high, the model may overestimate the decomposition and accumulation rate of SOC during the simulation process.	[33]
Roth C^[^ ^103^ ^]^	26.3	4‐5^[^ ^31^ ^]^	Clay content, organic carbon, bulk density, inert organic matter	Monthly total precipitation, maximum/minimum average temperature, and total evaporation	Crop residue quality, residue carbon input, manure carbon inputs, soil cover (bared or covered by vegetation)	Does not include carbon input from plant growth and requires obtaining carbon input data from other models or data sources (lack of dynamic coupling between plant growth and SOC). Low performance under stubble management scenarios, resulting in overestimation of SOC content. Not suitable for double rice cropping plots (because of the high amount of organic matter inputs there).	[104]
Yasso07^[^ ^105^ ^]^	07	5^[^ ^33^ ^]^	Initial organic carbon content	Annual average precipitation, maximum/minimum average temperature	Litter and biomass	Based on measurable chemical pools rather than steady‐state assumptions, thus relies on key parameters such as decomposition rates and transformation rates of organic carbon pools. In long‐term predictions, the uncertainty of these parameters can impact the results. Over time, these variations and prediction errors may compound.	[77]

Note: The first column of the table is the model name and model developer, the third column is the division of the carbon pool in the reference, and the last two column is the literature source of the model's disadvantages.

### 6.3 Trials and Tribulations of Biogeochemical Modelling

Many researchers assume that modelling workflows, encompassing initialization, calibration, validation, and data selection, are inherently consistent and, as such, often omit explicit documentation of these processes, potentially perceiving them as self‐evident. However, inconsistencies in workflow implementation or inadequate documentation can compromise the scientific rigor and defensibility of simulated SOC outcomes. A key example is inconsistent initialization methods between validation and project modelling.^[^ ^79^ ^]^ If different initialization approaches are used at these stages, the validation process may not accurately reflect the actual project modelling procedure, leading to discrepancies in model performance. Despite its importance, SOC pool initialization is often not explicitly addressed in model operating manuals. Wiltshire et al.^[^ ^80^ ^]^ highlight that true SOC equilibrium is likely rare. Given the extensive land‐use changes of the 20th century and the long stabilization periods required for some SOC pools (e.g., humified material), SOC stocks are often in flux.^[^ ^81^ ^]^ Typically, SOC is either decreasing (e.g., conversion of forests to farmland) or increasing (e.g., forest succession or regenerative agriculture).^[^ ^82^ ^]^ Capturing these dynamics requires a specific initialization approach. First, it should generally account for historical land use changes. Second, it should involve an iterative process, where the model runs for an extended period (usually thousands of years) until the slower‐changing pools stabilize. During this phase, carbon inputs are adjusted based on post‐initialization SOC stocks, validated against empirical data.^[^ ^83^ ^]^ Lastly, one option is to use the default library distribution and initial sizes, or to empirically define the initial state of each library, as discussed above.

We highlight several other limitations that require attention in future research:

- **Lack of empirical data to populate models**: Biogeochemical models often rely on estimates, surrogate data, or generalized data from literature, especially in regions with scarce data or for specific soil types and land management practices. This can introduce significant parameter uncertainties.
- **Coarse model units**: Many models use large grid cells (e.g., 1 km^2^ or larger), which fail to capture the micro‐scale spatial variability of soil and crop properties. These microvariations can significantly impact carbon storage and nutrient cycling.
- **Limitations of profile‐scale models**: Profile‐scale models typically assume that conditions at a single location represent the entire region, thereby overlooking the spatial heterogeneity across the area. As a result, profile‐scale models cannot simulate spatial patterns at the regional scale.
- **Insufficient integration of microbial processes**: Advanced process dynamics, such as microbial processes, are often not fully or only partially integrated into many models.^[^ ^84^ ^]^ As a result, most simulation models are semi‐physically based, relying on equations with numerous empirical parameters that require re‐calibration.

We recommend that more effort be placed into comparison of how modelers model.^[^ ^33^ ^]^ For example, two users of the same model and application often result in different calibrations and validation statistics. This is because, while model sensitivity often guides the selection of parameters for calibration, many process‐based modelers subjectively make these decisions based on data availability rather than a deep understanding of model structure and ecosystem processes. For consistency in any given application, we recommend use of standardised protocols, with version control, and interoperability. Having a single operator may help ensure consistency, however, we believe that multiple operators with clear documentation and oversight should be involved to prevent bias and reduce the risk of errors. Collaboration and review across different users can help ensure consistant model calibration and validation processes.

To enhance the predictive capability of SOC models, we suggest:

1. Comparisons of how reliably each model simulates total SOC and SOC fractionation (particularly stable organic carbon fractions, such as mineral associated organic carbon, and labile fractions, such as particulate organic carbon) to identify robust processes and those requiring additional detail.
2. Conduct model sensitivity analyses to determine parameters that significantly influence model outcomes versus those that have minimal impact.
3. Default‐out insensitive parameters to reduce the parameterization burden on users and simplify model setup.
4. Identify and remove processes that contribute little to the results, enhancing model efficiency without compromising accuracy (model simplification).
5. Using an ensemble of existing models allows leveraging their collective strengths while accounting for management practices, climate, land‐use change, and extreme weather events.
6. Leveraging RS technology to broaden data sources (as discussed in 4. Sensing technologies for soil carbon prediction) and combining AI with biogeochemical models into a unified modelling system aims to enhance predictive accuracy and improve model transferability, although this requires more computational resources. The uncertainties of the biogeochemical modelling come from the model structure, model parameters, and model inputs. The RS datasets can be effectively integrated to generate model inputs with an aim to reflect the land management practices and carbon inputs into the system for large‐scale simulations.^[^ ^85^ ^]^

## 7 Integration Approaches

### 7.1 Integrating RS/Sensor Data and AI to Predict SOC

The integration of RS and AI offers a possible approach for estimating SOC.^[^ ^40c^ ^]^ RS‐derived environmental covariates, such as topographic attributes and vegetation indices, serve as inputs for AI models.^[^ ^17^ ^]^ However, the performance of these models depends on the covariates selection, the AI algorithms used, and the extent to which RS data are integrated. The choice of environmental covariates varies across studies. In some cases, covariates are selected based on expert knowledge. For example, Meliho et al.^[^ ^106^ ^]^ identified 24 climate variables, 12 topographic variables, and 15 RS‐derived variables as key SOC predictors in the Moroccan High Atlas. Other studies take a more data‐driven approach. Hengl et al.^[^ ^107^ ^]^ used 158 RS‐based soil covariates for model calibration, while Nguyen et al.^[^ ^108^ ^]^ relied exclusively on Sentinel‐1 and Sentinel‐2 data to enhance SOC prediction robustness. A systematic approach to feature selection was demonstrated by Xiong et al.^[^ ^68a^ ^]^ who compiled a comprehensive set of environmental covariates—STEP‐AWBH variables (S: Soil, T: Topography, E: Ecology, P: Parent Material, A: Atmospheric/climate, W: Water, B: Biota, and H: Human factors). They then used multiple selection methods (e.g., Boruta, greedy forward/backward selection, hill climbing, and simulated annealing) in combination with four ML algorithms (RF, Cubist, Bagged Regression Trees, Boosted Regression Trees) to identify an optimal and minimal predictor set. This process reduced an extensive set of 210 potential predictors to just four key variables.

Reducing the number of input variables improves model prediction quality, avoids overfitting, and enhancing prediction accuracy.^[^ ^23^, ^106^ ^]^ Wadoux et al.^[^ ^23^ ^]^ articulate two strategies: 1) pre‐selection of covariates based on statistical correlation (e.g., Pearson's r) before model calibration, which is suitable for regression tasks where both inputs and outputs are continuous, and 2) recursive feature elimination (RFE), which iteratively removes the least important features based on model performance. The suitability of RFE depends on the base estimator—it can be used for either regression or classification, depending on the algorithm selected (e.g., linear regression for regression tasks, or logistic regression for classification tasks). Beyond these, various feature selection methods have been developed to handle large and complex datasets. The Boruta algorithm is widely used in classification problems, as it identifies all relevant features by comparing them to randomized shadow features.^[^ ^109^ ^]^ SHAP, another method that originated from game theory, can be applied to both regression and classification tasks, and offers both variable importance ranking and interpretability of predictions.^[^ ^24^, ^110^ ^]^ VSURF (variable selection using RF) is another method designed for high‐dimensional data, applicable to regression and classification, and particularly effective when the number of predictors far exceeds the number of observations.^[^ ^111^ ^]^

In SOC prediction, multicollinearity — when independent variables in a linear regression equation are correlated — can increase parameter variance, lead to unstable coefficient estimates, and confound interpretation of variable importance.^[^ ^109^ ^]^ Feature selection methods, such as LASSO, Elastic Net and several regularization techniques are aimed at addressing multicollinearity by penalizing large coefficients and shrinking less informative predictors.^[^ ^112^ ^]^ Principal component analysis can also be invoked to reduce the complexity of variable analysis by selecting features that have the greatest impact on predictive performance.^[^ ^112^ ^]^ Integrating domain knowledge (e.g., known soil–climate interactions) with data‐driven methods can also improve the interpretatibility of SOC models.

Besides covariate selection, data imbalance remains a challenge in environmental modelling, especially when high or extreme observations are underrepresented in the training dataset. This issue is particularly prominent in SOC prediction across heterogeneous landscapes, as field sampling is often sparse or skewed. A relevant example outside the soil field comes from Asadollah et al.,^[^ ^113^ ^]^ who used a RF model to combine multi‐sensor Landsat reflectance data and dissolved organic carbon in lakes measurement data and addressed the problem of skewed data distribution through the synthetic minority oversampling technique (SMOTE). Their findings highlight that data imbalance can lead to systematic underestimation of environmental variables, while RS–based ML frameworks can be improved via data augmentation methods. While DOC and SOC are monitored in different ecosystems, the underlying problem of sample sparsity and data imbalance is shared. Therefore, such integrated approaches (RS + ML + SMOTE) provide a transferable framework for SOC modelling, especially when data are limited or sample distributions are skewed.

### 7.2 Integrating RS/Sensor Data and Biogeochemical Models to Predict SOC

Biogeochemical models are effective tools for quantifying SOC in ecosystems by providing an understanding of its dynamics. These models consider the influence of soil characteristics, crop types, and field management practices on SOC dynamic. However, their performance is often constrained by the complexity of the underlying mechanisms, which results in difficulties with their parameterization. At regional scales, the heterogeneity of surface and near‐surface environments further complicates the acquisition of macro‐level data and the regionalization of model parameters.

**Figure 10:**
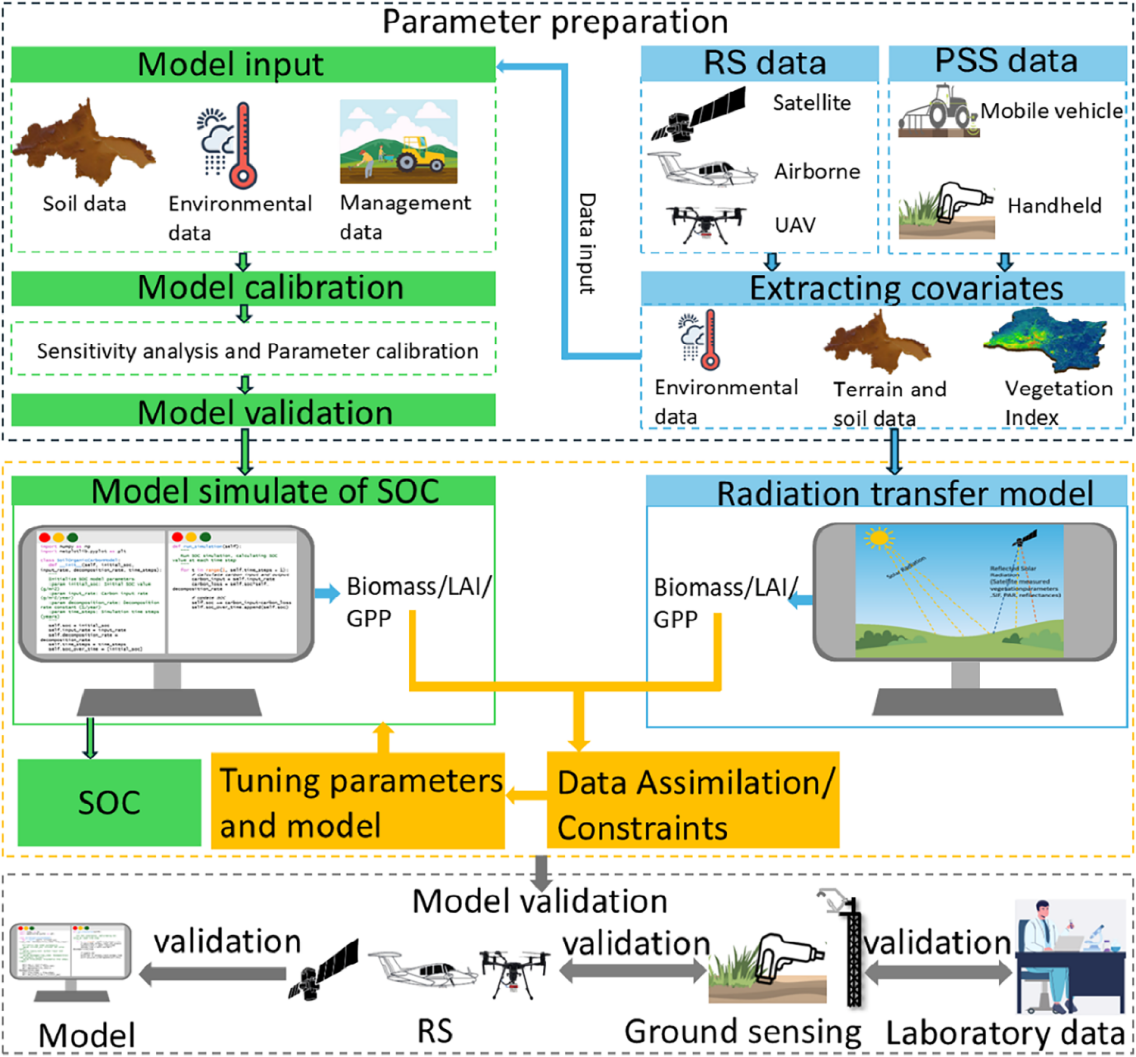
Workflow for integrating model simulations with remote sensing (RS) and proximal soil sensing (PSS) data for soil organic carbon (SOC) estimation. Diagram illustrates the process starting from parameter preparation, which includes model input data (soil, environmental, and management data) and data sourced from satellites, airborne platforms, unmanned Aerial Vehicles (UAVs) and PSS. Covariates are extracted using environmental data, terrain data, and vegetation indices. Model calibration involves sensitivity analysis and parameter tuning, leading to model validation. The SOC simulation process biomass, leaf area index (LAI) and generates gross primary production (GPP) outputs, which are refined through data assimilation and constraints. The radiation transfer model also computes GPP using RS inputs. Model performance is validated through comparisons with laboratory analysis data, RS data and ground sensing observations.

Integrating RS information into models through model‐data fusion technology (MDF) is a possible approach for enhancing model quality.^[^ ^48^, ^114^ ^]^ As shown in the MDF framework in **Figure** **10**, RS provides continuous temporal and spatial data on crop biophysical variables and SOC estimates, such as gross primary production (GPP) and leaf area index.^[^ ^115^ ^]^ These observations can be used to adjust model parameters, state variables, or structures to improve predictions. Research on integrating RS and biogeochemical models typically employs two main approaches: forcing and assimilation.^[^ ^116^ ^]^ The forcing method directly substitutes model parameters with values derived from RS data, driving the model's operation. Data assimilation, on the other hand, updates model simulations by incorporating the best constraints derived from estimated measurements and model prediction errors, so that the model state aligns with observed results. Ye et al.^[^ ^114^ ^]^ used the MDF method to combine remotely sensed cover crop biomass data with process models to improve the prediction accuracy of SOC. Specifically, they constrained and validated process‐based models using aboveground biomass data obtained from satellite RS. RS‐constrained models significantly improved the quantification of aboveground biomass carbon in cover crops, increased R^2^ from 0.60 to 0.87 compared with unconstrained simulations. It is an expected results since aboveground biomass serves as a major carbon source for SOC.

Rapid advancements in RS technology have addressed many practical challenges in modelling, driven by continuous product development and increased digital data availability. Nevertheless, in the era of “big data”, modellers often encounter difficulties in selecting suitable datasets, as different data sources can vary significantly in accuracy and resolution. Further, not all data contribute to better model performance. In some cases, their integration may introduce redundancy or errors. Evaluating and comparing datasets from different sensors, spatial scales, retrieval methods, and resolutions can help identify the most suitable inputs for carbon cycle modelling. Additionally, validating predictions through independent datasets (such as in situ observations) remains essential.

### 7.3 Ensemble Models: Combining Multiple AI Algorithms or Multiple Biogeochemical Models

Ensemble modelling (i.e., the integration of multiple different AI algorithms or multiple different biogeochemical models) has been recognized as a strategy to SOC predictions. In the field of AI, averaging or weighting the predictions of various AI algorithms can reduce model‐specific errors and improve overall predictive performance. Sun et al.^[^ ^22^ ^]^, for example, showed that combining RF, SVM, XGBoost, and ANN improved predictions (RMSE = 1.29, R^2^ = 0.85) compared to using single models (RF: RMSE = 2.05, R^2^ = 0.62; SVM: RMSE = 2.08, R^2^ = 0.61; XGBoost: RMSE = 2.39, R^2^ = 0.48; ANN: RMSE = 2.64, R^2^ = 0.37). Tran et al.^[^ ^117^ ^]^ also found that combining multiple ML algorithms into an ensemble model improved SOC prediction (R^2^ = 0.76, RMSE = 0.66). Zeraatpisheh et al.^[^ ^118^ ^]^ constructed an ensemble model that used a weighted average of SVM, RF, ANN, and k‐Nearest Neighbor, which outperformed all individual models (ensemble model R^2^ = 0.35, while the R^2^ of individual models was below 0.15). Measuring the central tendency of results from different algorithm models can reduce uncertainty by balancing the errors of each model, thereby achieving a better fit.^[^ ^33^ ^]^

Similarly, ensemble frameworks have also been applied to biogeochemical models by averaging or weighting the outputs from multiple process‐based simulations to account for structural uncertainties and model‐specific sensitivities. For example, Farina et al.^[^ ^31^ ^]^ evaluated the performance of 26 biogeochemical models in simulating long‐term SOC dynamics under bare fallow conditions. They compared model outputs with observational data from six long‐term bare fallow sites across Europe, and found that ensemble modelling improved the SOC predictions under different calibration strategies (R^2^ = 0.937 for scenario‐blind simulations, and R^2^ = 0.994 for site‐specific calibrated simulations). Tebaldi and Knutti et al.^[^ ^119^ ^]^ also emphasized that combining multiple simulations tends to enhance prediction accuracy, especially when overall model performance is considered. Farina et al.^[^ ^31^ ^]^ suggested that a minimum of 10 models may be required for ensemble predictions when calibration is not possible, while as few as 3 to 4 models may suffice when site‐specific calibration is feasible. Still, this threshold may depend on the degree of structural diversity among the models included in the ensemble. Process‐based models differ in parameter settings and structures for core processes such as carbon input, decomposition rate, and stable carbon pools. Integrating these models into an ensemble can make up for the shortcomings of a single model in characterizing a specific process and reveal the reasons for the differences in predictions between models.

The challenges of ensemble modelling lie in the lack of clear standards for model selection and combination, making it difficult to ensure complementarity among sub‐models. Additionally, parallel computation of multiple models incurs high computational costs, particularly in large‐scale applications. Incorrect ensemble strategies may fail to correct the biases of individual models. In the future, establishing standardized benchmarking platforms to systematically evaluate the performance of different ensemble strategies in typical scenarios could help optimize model selection and combination strategies. Meanwhile, as our understanding of SOC processes changes, new mechanisms can be gradually introduced or redundant processes streamlined.

### 7.4 Hybrid models: Combining RS/Sensor Data, AI, and Biogeochemical Models

**Figure 11:**
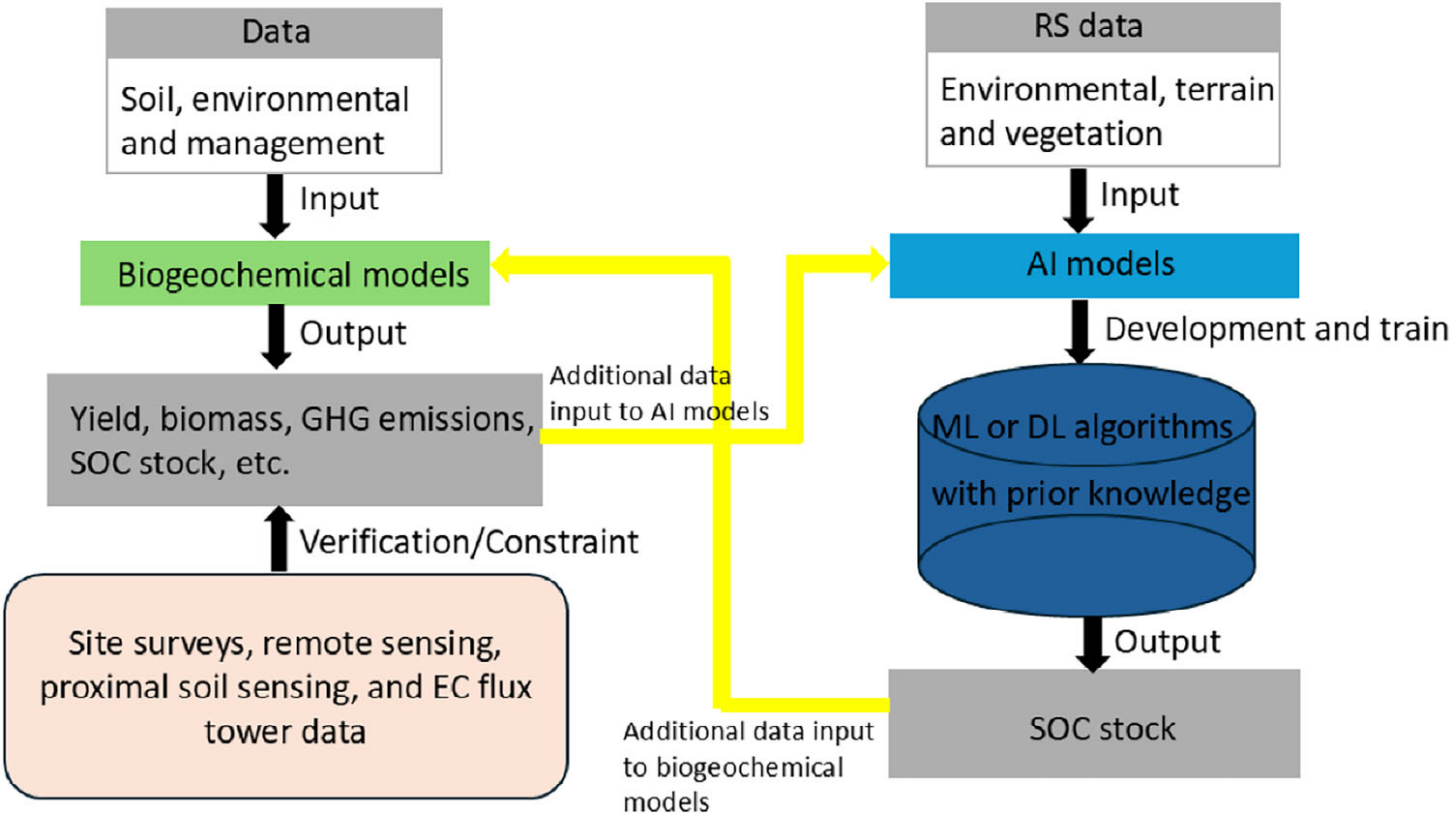
Concept figure of the integration model. The simulation results based on the biochemical model serve as additional training data for the artificial intelligence (AI), and the developed AI is guided by prior knowledge. The reverse hybridization is AI models produce soil organic carbon (SOC) predictions that are then streamed into biogeochemical models. Site surveys, remote sensing (RS), soil proximal sensing (PSS) and eddy covariance (EC) data are used to verify and constrain the model results.

SOC modelling hinges upon how well we understand and emulate fundamental soil science processes, as well as how such science is represented in modelling processes. All models are simplifications of reality; if they were not simplifications, they would not be models but would be reality. The question of which processes should be captured and which omitted depends on the purpose for which the model has been derived. The majority of AI‐based SOC predictions are used in static scenarios (i.e. one model for a prediction at one time step), which result in strong interannual fluctuations in time series SOC ^[^ ^120^ ^]^ when the objective is space and time prediction. DL methods, such as Long‐Short Term Memory (LSTM) and CNN, can model space‐time sequences of soil and ecosystem properties. LSTM has been applied to model soil hydrology using RS‐informed modelling.^[^ ^121^ ^]^ However, applying DL algorithms to model SOC sequestration is somewhat problematic due to lack in long‐term experimental field plots and SOC monitoring programs. Biogeochemical models capture SOC dynamics explicitly through mechanisms as described in Table 2. Nevertheless, these models require substantial effort to understand and parameterize the SOC turnover processes. Their complexity demands extensive input data, and gaps in knowledge or data availability can lead to structural errors.^[^ ^122^ ^]^ In practice, AI and biogeochemical models can effectively complement each other. Incorporating simulation outputs from biogeochemical models as additional training data for ML can support conscious modelling of SOC turnover processes while retaining some of the spatial prediction accuracy of AI. To this end, as shown in **Figure** **11**, we conceptualize the hybrid model. In addition, RS observations with its temporal repetition and broad spatial coverage may provide inputs for both types of models, which may in turn support model verification and add some constraints.^[^ ^40c^ ^]^

The hybrid approaches have been widely used. Zhang et al.^[^ ^123^ ^]^ demonstrated this by integrating two process‐based models (Roth‐C and microbial‐mineral carbon stabilization, MIMICS) with RF. Outputs from the process models were used as dynamic covariates within the space‐for‐time random forest model to simulate the temporal variation of SOCso as to capture the spatiotemporal dynamics of SOC. The hybrid model including Roth‐C + MIMICS + RF improved R^2^ by 80% and 59% compared with the single model of Roth‐C and MIMICS, respectively.^[^ ^123^ ^]^ Hybrid models can also be used for mismatched spatial/temporal resolutions and computations limitations. For example, Zhang et al.^[^ ^35a^ ^]^ proposed an integration framework that used SOC data simulated by process models as additional training data, combined with actual measured SOC data to train AI models. The introduction of simulated process model outputs improves the temporal representativity of training samples, particularly for years lacking field data. As a result, the AI can leverage both existing sampling data and dynamic predictions provided by the process model in data‐sparse periods. Of particular note is the knowledge‐guided machine learning (KGML) model framework proposed by Liu et al.,^[^ ^35b^ ^]^ which integrates process‐based model (i.e. the ECOSYS model) with DL to improve the prediction accuracy of agricultural carbon cycling. ECOSYS generates synthetic data for key variables like crop yield, Ra, Rh, NEE, and GPP, which are used to pre‐train the KGML‐ag‐Carbon model. Pre‐training enables the model to better capture the dynamic changes in the carbon cycle. Additionally, RS observations of GPP, representing the primary carbon input in agricultural ecosystems, are incorporated as spatial constraints. Finally, the model is fine‐tuned and validated using observed data (e.g., from EC and chambers). These case studies indicate that the hybrid approach successfully combines the strengths of biogeochemical models and AI, compensating for the limitations of individual models in spatial or temporal predictions. Such hybrid models address challenges posed by low sampling density in space and time by expanding the training data. In these studies, the causal relationships of ecological processes captured by the process‐based models are combined with data‐driven prediction. For these reasons, hybrid models improve the spatial and temporal SOC prediction quality, but also support interpretability.^[^ ^35^ ^]^ In situations with data scarcity or discontinuous observations, datasets generated from process models can be used for pre‐training AI models, providing prior knowledge.^[^ ^35b^ ^]^ Moreover, these datasets are much less costly than large‐scale field observations, thereby expanding the spatial coverage of training samples and enhancing the model's generalization ability over time. This is because many process‐based models are driven by climate data, and climate measurements tend to be more ubiquitous than measurements of SOC at scale.

Development of hybrid models for SOC prediction remains in its infancy. Existing SOC process models are quasi‐physical and semi‐empirical at best.^[^ ^92a^ ^]^ Before developing a new biogeochemical process‐based model, it is essential to understand and mathematically encapsulate the complex turnover mechanisms and causal relationships driving SOC dynamics.^[^ ^84^ ^]^ The design and integration of the model must be tailored to the specific conditions of the target region, as SOC pathways vary widely depending on soil types, parent materials, climate scenarios, and land management practices.^[^ ^124^ ^]^ For instance, differences in farming methods and moisture conditions influence carbon decomposition rates and soil formation processes, requiring models to be adjusted accordingly.^[^ ^15a^ ^]^ Similarly, management practices such as irrigation, fertilization, and tillage play significant roles in shaping the pathways of SOC change and must be incorporated into the model.^[^ ^15a^ ^]^ Spatiotemporal dynamics of SOC are governed by a combination of natural and anthropogenic factors, as well as a range of biophysical processes, making it challenging to fully encapsulate these dynamics within a single model. As a result, model design requires a careful balance between complexity and parsimony, with intentional decisions on which factors to include and which to omit to maintain both accuracy and simplicity.

## 8 Recommendations for Advancing the State of the Art

Future soil carbon research is expected to benefit from technological and methodological advancements. This can be fuelled by the digital convergence.^[^ ^125^ ^]^ Here we outline strategic recommendations informed by current challenges and advancements in SOC modelling. These recommendations aim to inform future research directions that can foster advancements in SOC modelling.

1. **Enhanced data collection for model training**

The limited availability of measured SOC data remains a constraint to model accuracy. There is a need to increase the deployment of flux towers, continuous high‐resolution data on carbon fluxes between the soil and the atmosphere. Such data are needed to understanding the dynamic exchange of carbon. Besides, better relationships between the diverse sensing technologies such as PSS and RS need to be developed to help obtain cost‐effective a spatially explicit data to calibrate AI and process‐based models. All in all, these new data can support calibration and validation of SOC models by providing complementary data streams.

2. **Selection of the optimal biogeochemical model**

The selection of an appropriate biogeochemical model for SOC simulation depends on the specific ecosystem being studied and the research objectives. No single model universally outperforms others, as each exhibits varying levels of adaptability to different environmental conditions. Variability in the quality of input data, such as meteorological and soil properties, parameter settings, and the availability of historical management data, introduce uncertainties into model outcomes. To mitigate these uncertainties, we need to develop modelling strategies based on coherent and standardized workflow, particularly during the stages of initialization, calibration, validation, and data selection. Workflows are also necessary in pre‐application testing to optimize model inputs and support collecting the right dataset.

3. **Integrating AI and biogeochemical models with big data**

We highlighted that the fusion of AI with biogeochemical modelling holds potential for improving SOC predictive modelling. This integration can support modelling with large‐scale and diverse environmental data collected through advanced sensing technologies. It can also support hybridization of AI and process models with cross‐fertilization of the SOC data or prediction being shared or streamed from one model type into the other. Yet, other hybridized model applications using tight‐ or loose coupling among models could be envisioned such as a) reverse engineering of process‐based equations via AI, b) learning and loss functions in AI models being coupled to a process‐based model, c) PSS monitoring data and spectral SOC predictions coupled to AI or biogeochemical models, and d) meta‐modelling or surrogate modelling of complex biogeochemical models with AI.

**4. Fostering multi and trans‐disciplinary collaboration**

Advancing SOC prediction requires collaboration among experts from diverse fields such as soil science, microbiology climatology, hydrology, ecology, agronomy, and computer science. Challenges such as overfitting, ambiguous model interpretations, and limited causal inference demand input from soil scientists, agronomists, and ecologists to guide model development and evaluation. Close coordination with stakeholders is critical to define acceptable generalization thresholds and identify application‐specific needs for interpretability and causal insights. Model developers and domain experts must work together to create and assess benchmark datasets, define evaluation criteria, and develop hybrid or physically constrained ML models. This collaborative approach will ensures model transparency, supports realistic uncertainty quantification and visualization, and fosters trust and usability in real‐world agricultural soil carbon decision‐making contexts (e.g. in soil carbon crediting schemes), aligning with similar efforts in other research domains.^[^ ^126^ ^]^

## 9 Conclusion and Limitations

Prediction of SOC must be grounded on mechanistic understanding of soil processes in concert with accuracy in simulation of complex spatiotemporal patterns. Biogeochemical models simulate SOC dynamics based on known processes, offering interpretability but facing challenges like input data demands and parameter uncertainty. They tend to be driven by climate data and point‐based, and thus suffer limitations in capturing spatial variability. In contrast, AI models excel in capturing spatial patterns but do not predict well temporal trends because of the lack of such datasets. By combining the temporal mechanistic strengths of process‐based models with the spatial pattern recognition abilities of AI, hybrid approaches embed causal knowledge and improve prediction, especially when training data are sparse. Synthetic data from process models supports AI model pre‐training, while high‐quality observations enable direct learning. Integrating multi‐source sensor data further enhances model input and validation and improv scalability.

Nonetheless, this review has several limitations. First, although we endeavoured to cover key methodological aspects, this review was not conducted under a formal systematic framework like PRISMA or SALSA, and some relevant literature may have been missing. Second, the comparison of model performance was primarily based on published case studies, which vary in geographical scale, data quality, and evaluation metrics—limiting direct comparability. Lastly, integration strategies discussed remain largely conceptual; further empirical testing and benchmarking are needed to validate their practical effectiveness across diverse soil systems.

## Conflict of Interest

The authors declare no conflict of interest.

## Supporting information



Supporting Information
