# Supplementary material for: Advancing Soil Organic Carbon Prediction: A Comprehensive Review of Technologies, AI, Process‐Based and Hybrid Modelling Approaches
Source: Adv Sci (Weinh). 2025 Jun 25;12(31):e04152. doi: 10.1002/advs.202504152 (PMC12376622; doi:10.1002/advs.202504152)
Supplement: Supplementary file 1 — Supporting Information [file ADVS-12-e04152-s001.docx]

Advancing soil organic carbon prediction: a comprehensive review of technologies, AI, process-based and hybrid modelling approaches

**Text S1.**

**Factors driving SOC stocks and fluxes**

Climate variables, particularly temperature and precipitation, are recognized as primary determinants of SOC storage at regional and global scales.^[1]^ Soil temperature governs microbial activity and soil respiration, influencing organic matter decomposition rates. While moderate increases in temperature generally enhance decomposition, extreme heat and cold can suppress microbial activity, leading to reduced SOC turnover (Figure. 1).^[2]^ Precipitation largely governs net primary productivity, which determines the magnitude of organic carbon inputs to the soil. Although humid conditions generally favour SOC sequestration, excessive soil moisture, such as that caused by waterlogging, can accelerate SOC losses through enhanced microbial decomposition and increased greenhouse gas (GHG) emissions, particularly methane.^[1a,2,3]^ In flooded agroecosystems (e.g., paddy fields), prolonged saturation, especially during early growth stages or excessive rainfall events, can lead to significant nutrient losses through leaching and denitrification, particularly of nitrogen. These losses not only reduce fertilizer use efficiency, but may also represent a greater threat to crop productivity and soil health than GHG emissions.^[4]^ The distribution of SOC is spatially heterogeneous and strongly influenced by topography.^[5]^ Steep slopes are particularly vulnerable to soil erosion, which facilitates the loss of SOC-rich surface layers, whereas lower slopes or depressions may act as deposition zones, promoting SOC accumulation.^[5,6]^ The physicochemical properties of soil parent material, including mineral composition, aeration, and permeability, regulate SOC stabilization processes. Parent material influences organic matter mineralization, humification efficiency, and nutrient availability, all of which contribute to variations in SOC turnover and storage.^[1a,7]^

Human interventions, particularly in agricultural landscapes, exert significant control over SOC dynamics, often surpassing the impact of natural factors. Indeed, human-induced SOC accrual due to practice change (as distinct from background changes in SOC due to climate) are the primary objective of many contemporary carbon crediting schemes, such as those of the Australian Government, Verra and Gold Standard. The effectiveness of management practices in enhancing SOC sequestration is, however, contingent upon climatic conditions and the frequency of extreme weather events.^[8]^ Agricultural management influences SOC fluxes through its impact on organic matter inputs, microbial activity, and SOC permanence. Diversified cropping systems, such as crop rotations, have been demonstrated to enhance SOC storage relative to monocropping systems by increasing belowground carbon inputs and improving soil structural stability.^[1a, 9]^ Other practices also play key roles in SOC variation, such as frequency and depth of cultivation, nutrient addition and residue retention. ^[10]^ Sustainable management practices that increase organic carbon inputs (e.g., crop residue retention, organic amendments, effective irrigation) and mitigate carbon losses (e.g., conservation tillage, reduced soil disturbance) are integral to improving SOC stocks.^[10]^ For example, Yuan et al.^[11]^ showed that combining residue retention with reduced tillage increased SOC by 13%. Sun et al.^[12]^ reported that flooding irrigation improved crop biomass and residue return, increasing SOC by 16%. Adoption of such strategies is increasingly emphasised in climate-smart agriculture initiatives and carbon crediting schemes aimed at enhancing soil carbon stocks while promoting long-term agroecosystem sustainability.

**Text S2.**

Landsat-8 provides medium-resolution multispectral (10–30 m) and thermal infrared (60 m) data, but its 16-day revisit cycle limits cloud-free image acquisition. Sentinel-2 offers a finer 10 m resolution with a shorter 5-day revisit period, improving monitoring capabilities, particularly in cloud-prone regions. Despite variable sensor characteristics, the Landsat program provides amongst the longest time-series datasets, dating back to 1972.

Sentinel-2's MSI includes 13 spectral bands, notably four narrow red-edge bands, such as B5 (698-713 nm), B6 (733-748 nm), B7 (773-793 nm) and B8a (855-875 nm), which have been invoked for SOC estimation due to their sensitivity to vegetation and soil conditions.^[13]^ While Sentinel 1 provides synthetic aperture radar (SAR) datasets with an active sensor that enabling collection of datasets despite the presence of cloud cover, accuracy of SAR in isolation, and approaches aimed at fusing SAR and optical data, have room for improvement. ^[14]^

Open-access RS platforms such as the Google Earth Engine facilitate multi-source data integration for SOC estimation. While satellite data offer large-scale monitoring, airborne and UAV-based RS provide higher spatial resolution and flexible flight planning for intra-field or regional studies.^[15]^ However, airborne hyperspectral imaging is susceptible to weather and incurs higher operational costs. UAV-based RS, in contrast, offers adaptability, lower costs, and ultra-high spatial resolution, but is suitable for smaller areas and may not be as amenable to the frequency used in satellite derived RS.^[16]^

**Literature Search Methods**

**Text S3.**

We conducted a data retrieval from the Web of Science using the keywords "*soil organic carbon*" and "*remote sensing*" and *predic**. A total of 279 papers matching these criteria were manually curated by reviewing their titles, abstracts, and keywords.

**Text S4.**

We retrieved data from the Web of Science using the keywords "*soil organic carbon*" and "*artificial intelligence*" and “*machine learning*” and "*deep learning*". By reading the "title", "abstract", and "method" section, we counted the frequency of use of AI algorithms in the literature. Fig. 5 shows our statistical results.

In the meta-analysis of **5.2** (Fig. 6), we searched the algorithms again based on the results of Fig. 5. First, the following search formula was used to search the literature:

(TS=(soil organic carbon)) AND (TS=(machine learning)) AND (TS=(random forest) OR TS=(RF)) AND (TS=( Support Vector Machine) OR TS=(SVM)).

According to the top 3 algorithms, after searching RF and SVM, the search formula was used to search RF and Cubist, SVM and Cubist in turn. According to the best results produced by RF, RF and ensemble (hybrid) models were searched.

By reviewing the "Method" section of each literature one by one, it was determined whether coefficient of determination (R²) and root mean square error (RMSE) were used as indicators to evaluate the performance of ML algorithms. Studies that included the use of these two evaluation indicators were included in the meta-analysis. Then we manually recorded the R² and RMSE values of the ML algorithms from each published study and used these data for subsequent statistical analysis.

Meta Win2.1 software was used for meta-analysis.^[17]^ This study used a random effects model to calculate the mean effect value, and the 95% confidence interval (CI) was estimated using the bootstrapping (n=4999) method. The effect values in this study were expressed as percentages and converted using the formula (e^lnR–1)×100%. If the 95% CIs did not overlap, the mean effect size was considered to be significantly different. In the example on the left side of the Fig.6 (RF vs. SVM), if the effect value is on the right side of the zero scale, it means that the R2 or RMSE of RF is significantly higher than that of SVM, and on the left side, it means that RF is significantly lower than SVM.

**Text S5.**

We searched the Web of Science database using phrases “*soil organic carbon*”, “*process-based model*”, and “*biogeochemical model*”. By reading the abstracts of the retrieved literature, we manually screened out 30 to 40 models used for SOC prediction (Fig. 7). Subsequently, we performed additional searches combining the names of selected models (e.g. “*Century*”) with “*soil organic carbon*” as keywords. For each valid result, we compiled annual publication records (Fig. 7).

**References**

1. a) M. Wiesmeier, L. Urbanski, E. Hobley, et al., Soil organic carbon storage as a key function of soils - A review of drivers and indicators at various scales, Geoderma **2019**, 333, 149-162; <http://doi.org/10.1016/j.geoderma.2018.07.026> b) Y. Yang, J. Ti, J. Zou, Optimizing crop rotation increases soil carbon and reduces GHG emissions without sacrificing yields, Agric. Ecosyst. Environ. **2023**, 342, 108220. <http://doi.org/10.1016/j.agee.2022.108220>
2. C.D. Koven, G. Hugelius, D.M. Lawrence, W.R. Wieder, Higher climatological temperature sensitivity of soil carbon in cold than warm climates, Nat Clim Change. **2017**, 7, 817-822. <http://doi.org/10.1038/NCLIMATE3421>
3. a) K.M. Christie, R.P. Rawnsley, M.T. Harrison, R.J. Eckard, Using a modelling approach to evaluate two options for improving animal nitrogen use efficiency and reducing nitrous oxide emissions on dairy farms in southern Australia, Anim. Prod. Sci. **2014**, 54, 1960-1970; <http://dx.doi.org/10.1071/AN14436> b) K. Liu, M.T. Harrison, A. Ibrahim, et al., Genetic factors increasing barley grain yields under soil waterlogging, Food Energy Secur. **2020**, 9, e238. http://doi.org/10.1002/fes3.238
4. [G. Kaur](https://acsess.onlinelibrary.wiley.com/authored-by/Kaur/Gurpreet), [G. Singh](https://acsess.onlinelibrary.wiley.com/authored-by/Singh/Gurbir), [P.P. Motavalli](https://acsess.onlinelibrary.wiley.com/authored-by/Motavalli/Peter+P.), [K.A. Nelson](https://acsess.onlinelibrary.wiley.com/authored-by/Nelson/Kelly+A.), [J.M. Orlowski](https://acsess.onlinelibrary.wiley.com/authored-by/Orlowski/John+M.), [B.R. Golden](https://acsess.onlinelibrary.wiley.com/authored-by/Golden/Bobby+R.), Impacts and management strategies for crop production in waterlogged or flooded soils: A review, Agron. J. 2020, 112, 1475-1501. <https://doi.org/10.1002/agj2.20093>
5. F. Bilotto, R. Vibart, A. Mackay, C. Des, M.T. Harrison, Towards an integrated phosphorus, carbon and nitrogen cycling model for topographically diverse grasslands, Nutr. Cycl. Agroecosyst. **2022**, 124, 153-172. <http://doi.org/10.1007/s10705-022-10231-3>
6. S. Doetterl, A.A. Berhe, E. Nadeu, Z. Wang, M. Sommer, P. Fiener, Erosion, deposition and soil carbon: A review of process-level controls, experimental tools and models to address C cycling in dynamic landscapes, Earth-Sci. Rev. **2016**, 154, 102-122. <http://doi.org/10.1016/j.earscirev.2015.12.005>
7. S.E. McDonald, W. Badgery, S. Clarendon, et al., Grazing management for soil carbon in Australia: A review, J. Environ. Manage. **2023**, 347, 118146. <http://doi.org/10.1016/j.jenvman.2023.119146>
8. A. Muleke, M.T. Harrison, R. Eisner, et al., Clarifying confusions over carbon conclusions: antecedent soil carbon drives gains realised following intervention, Global Environ. Change Adv. **2023**, 1, 100001. <https://doi.org/10.1016/j.gecadv.2023.100001>
9. a) L.K. Tiemann, A.S. Grandy, E.E. Atkinson, E. Marin-Spiotta, M.D. McDaniel, Crop rotational diversity enhances belowground communities and functions in an agroecosystem, Ecol. Lett. **2015**, 18, 761-771; <http://doi.org/10.1111/ele.12453> b) E.A. Meier, P.J. Thorburn, L.W. Bell, M.T. Harrison, J.S. Biggs, Greenhouse gas emissions from cropping and grazed pastures are similar: a simulation analysis in Australia, Front. Sustain. Food Syst. **2020**, 3, 1-18. <https://doi.org/10.3389/fsufs.2019.00121>
10. a) T. Ramesh, N.S. Bolan, M.B. Kirkham, et al., Soil organic carbon dynamics: Impact of land use changes and management practices: A review, Adv. Agron. **2019**, 156, 1-107; http://doi.org/10.1016/bs.agron.2019.02.001 b) M.A. Khan, A. Basir, S. Fahad, et al., Biochar optimizes wheat quality, yield, and nitrogen acquisition in low fertile calcareous soil treated with organic and mineral nitrogen fertilizers, Front. Plant Sci. **2022**, 13, 879788. <https://doi.org/10.3389/fpls.2022.879788>
11. Q. Yuan, H. Shen, T. Li, et al., Deep learning in environmental remote sensing: Achievements and challenges, Remote Sens. Environ. **2020**, 241, 111716. <https://doi.org/10.1016/j.rse.2020.111716>
12. W. Sun, Z. He, D. Ma, B. Liu, R. Li, S. Wang, A Malekian, Response of soil carbon and nitrogen stocks to irrigation - A global meta-analysis, Sci. Total Environ. **2024**, 957, 177641. <https://doi.org/10.1016/j.scitotenv.2024.177641>
13. a) J. Segarra, M.L. Buchaillot, J.L. Araus, S.C. Kefauver, Remote sensing for precision agriculture: sentinel-2 improved features and applications, Agronomy **2020**, 10, 641; <http://doi.org/10.3390/agronomy10050641> b) L. Guo, P. Fu, T. Shi, Y. Chen, C. Zeng, H. Zhang, S. Wang, Exploring influence factors in mapping soil organic carbon on low-relief agricultural lands using time series of remote sensing data, Soil Tillage Res. **2021**, 210, 104982. <http://doi.org/10.1016/j.still.2021.104982>.
14. M.G. Ogungbuyi, C. Mohammed, I. Ara, A.M. Fischer, M.T. Harrison, Advancing Skyborne Technologies and High-Resolution Satellites for Pasture Monitoring and Improved Management: A Review, Remote Sens. **2023**, 15, 4866. <https://www.mdpi.com/2072-4292/15/19/4866>
15. a) E. Vaudour, J.M. Gilliot, L. Bel, J. Lefevre, K. Chehdi, Regional prediction of soil organic carbon content over temperate croplands using visible near-infrared airborne hyperspectral imagery and synchronous field spectra, Int J Appl Earth Obs Geoinf. **2016**, 49, 24-38; http://doi.org/10.1016/j.jag.2016.01.005 a) L. Guo, X. Sun, P. Fu, Mapping soil organic carbon stock by hyperspectral and time-series multispectral remote sensing images in low-relief agricultural areas, Geoderma **2021**, 398, 115118; <http://doi.org/10.1016/j.geoderma.2021.115118> c) Y. Hong, S. Chen, Y. Chen, et al., Comparing laboratory and airborne hyperspectral data for the estimation and mapping of topsoil organic carbon: Feature selection coupled with random forest, Soil Tillage Res. **2020**, 199, 104589. <http://doi.org/10.1016/j.still.2020.104589>
16. M. Wehrhan, M. Sommer, A parsimonious approach to estimate soil organic carbon applying unmanned aerial system (UAS) multispectral imagery and the topographic position index in a heterogeneous soil landscape, Remote Sens. **2021**, 13, 3557. <http://doi.org/10.3390/rs13183557>
17. M.S. Rosenberg, D.C. Adams, J. Gurevitch, **2000**, MetaWin-statistical Software for Meta-analysis. Sinauer Associates Inc., Sunderland. https://www.metawinsoft.com/
